# Beyond Oncology: Exploring the Expanding Role of CAR T Cell Therapy in Autoimmune and Infectious Diseases—A Systematic Review

**DOI:** 10.3390/pathophysiology33030070

**Published:** 2026-09-19

**Authors:** Kawther Zaher, Jehan Alrahimi

**Affiliations:** 1 Neuroscience and Geoscience Unit, King Fahd Medical Research Center, King Abdulaziz University, Jeddah 22254, Saudi Arabia; 2 Department of Medical Laboratory Sciences, Faculty of Applied Medical Sciences, King Abdulaziz University, Jeddah 22254, Saudi Arabia; 3 Department of Biological Sciences, Faculty of Sciences, King Abdulaziz University, Jeddah 22254, Saudi Arabia; 4 Immunology Unit, King Fahd Medical Research Center, King Abdulaziz University, Jeddah 22254, Saudi Arabia

**Keywords:** CAR T cells, chimeric antigen receptor, autoimmune disease, systemic lupus erythematosus, infectious disease, HIV, hepatitis B, CAR-Treg, immune reset, cellular therapy

## Abstract

**Background:** Chimeric antigen receptor (CAR) T cell therapy has revolutionized the treatment of select hematologic malignancies and is increasingly being explored for non-oncological indications, including autoimmune diseases and persistent infectious diseases. This systematic review synthesized current evidence on CAR T cell and CAR-Treg approaches beyond oncology, with emphasis on therapeutic targets, translational and clinical outcomes, safety, and implementation barriers. **Methods:** The review followed PRISMA 2020 guidelines. PubMed/MEDLINE, Scopus, Web of Science, Cochrane Library, ClinicalTrials.gov, and major trial registries were searched for studies published from 1 January 2010 to 31 March 2026. Eligible studies included clinical, preclinical, and translational investigations of CAR T cell or CAR-Treg strategies in autoimmune or infectious diseases. Results: Because of heterogeneity in disease indications, CAR constructs, endpoints, and study designs, findings were synthesized descriptively. The strongest early clinical signals were observed with B cell-directed CAR T cell therapy for severe, refractory autoimmune diseases, particularly systemic lupus erythematosus, systemic sclerosis, and inflammatory myopathies. Infectious disease applications, mainly HIV and hepatitis B, showed preliminary safety, persistence, and partial antiviral activity, but durable pathogen eradication remains unproven. Conclusion: Overall, CAR T cell therapy beyond oncology is promising but remains preliminary, requiring standardized reporting, long-term safety monitoring, scalable manufacturing, and carefully defined risk–benefit thresholds. Human clinical evidence was interpreted separately from preclinical and mechanistic evidence, and clinical conclusions were based primarily on human studies.

## 1. Introduction

Chimeric antigen receptor (CAR) T cell therapy is one of the most significant achievements in modern cellular immunotherapy. Its central innovation is to redirect T lymphocytes to specific surface antigens via synthetic receptors that couple an extracellular binding domain to the intracellular T cell activation machinery. Unlike classical T cell receptor recognition, CAR recognition is independent of peptide presentation by major histocompatibility complex molecules, enabling engineered T cells to identify target cells directly based on surface antigen expression [1]. This feature has made CAR T cells particularly effective against B cell malignancies, in which lineage antigens such as CD19 and BCMA (B cell maturation antigen) can be therapeutically targeted [2,3,4,5]. The same feature now provides the conceptual foundation for using CAR T cells beyond oncology, where pathogenic immune cells, autoreactive compartments, infected cells, or tissue-specific inflammatory niches may be selectively targeted.

The success of CAR T therapy in hematologic malignancies did more than establish a new cancer treatment; it validated a programmable immune-engineering platform [6,7]. First-generation CARs provided CD3-zeta signaling but were limited by poor expansion and persistence. Second-generation constructs added costimulatory domains such as CD28 or 4-1BB, improving in vivo proliferation, durability, and antitumor efficacy. Third-generation and later constructs introduced additional costimulatory domains, cytokine payloads, switch systems, logic gates, or gene-editing modifications to refine potency and safety. These engineering principles are now being reinterpreted for non-malignant disorders, where the therapeutic goal is often immune recalibration rather than maximal cytotoxicity [8,9].

The current CAR-therapy landscape is broader than that of conventional single-chain variable fragment CAR T cells. Contemporary platforms include bicistronic CAR designs that co-express two antigen-recognition modules or accessory genes, tandem CARs that incorporate two binding domains in one receptor, logic-gated CARs designed to improve specificity, transient mRNA CARs intended to limit persistence, armored CARs that deliver immune-modulatory payloads, and allogeneic CAR products, including gene-edited ‘universal’ constructs (a subset of allogeneic products further engineered, e.g., by TCR/HLA knockout, to reduce immunogenicity and graft-versus-host risk), intended to shorten manufacturing time. CAR-NK cells and other non-T cell CAR platforms are also important in the wider field; however, they were discussed only for contextual comparison because the eligibility criteria of this systematic review were restricted to CAR T cell and CAR-Treg interventions [7,8,9].

Autoimmune diseases provide the clearest rationale for this expansion. Many severe autoimmune disorders are sustained by pathogenic B cells, autoreactive memory B cells, autoantibody-producing plasmablasts, antigen presentation, and inflammatory cytokine networks. Standard immunosuppressive therapies can reduce disease activity but rarely eliminate the autoreactive immune architecture responsible for relapse [10]. Monoclonal antibodies such as rituximab can deplete circulating CD20-positive B cells, but they may not fully target tissue-resident B cells, early B-lineage populations, plasmablasts, or disease-sustaining lymphoid niches. By contrast, CAR T cells are living drugs capable of expansion, trafficking, serial killing, and deep tissue penetration. This creates the possibility of a more profound immune reset, in which pathogenic B cell memory is erased, and immune reconstitution occurs from a less autoreactive repertoire [11,12].

Systemic lupus erythematosus (SLE) has become the flagship autoimmune indication for this concept. Severe refractory SLE is marked by systemic inflammation, autoantibody production, complement consumption, immune complex deposition, and organ-threatening manifestations, including lupus nephritis. Early clinical reports of CD19-directed CAR T cell therapy in selected patients with severe refractory SLE have described drug-free remission, improvement in serological activity, disappearance or reduction in anti-double-stranded DNA antibodies, and recovery of complement levels; however, these findings require confirmation in larger controlled cohorts [13,14,15,16].

The infectious disease field approaches CAR T therapy from a distinct biological perspective. Chronic viral infections such as HIV and hepatitis B virus (HBV) persist despite effective antiviral drugs because of latent reservoirs, immune exhaustion, viral escape, tissue sanctuaries, and incomplete immune-mediated clearance. For HIV, antiretroviral therapy suppresses replication but does not eliminate the integrated provirus in long-lived cellular reservoirs [17,18,19,20]. For HBV, nucleos(t)ide analogs control replication but rarely eradicate covalently closed circular DNA or achieve a durable functional cure. CAR T cells could theoretically recognize conserved viral antigens on infected cells, bypass exhausted endogenous T cell responses, and deliver targeted cytotoxicity against reservoir-containing or antigen-expressing cells [21,22]. However, infectious indications also pose unique challenges, including low or intermittent antigen expression, viral mutation, HIV infection of T cells themselves, and tissue damage when infected cells reside in essential organs such as the liver [17,18,19,20].

A second major non-oncologic direction is the development of regulatory CAR T cells (CAR-Tregs). Unlike conventional cytotoxic CAR T cells, CAR-Tregs are engineered to suppress immune responses in an antigen-directed manner. This strategy is especially attractive for autoimmune disease, transplantation, allergy, inflammatory bowel disease, and neuroinflammation because it localizes immunosuppression to relevant tissues while preserving systemic host defense. CAR-Tregs combine the trafficking and specificity of engineered receptors with the suppressive biology of regulatory T cells, including cytokine-mediated suppression, metabolic disruption, cytolysis of effector immune cells, modulation of antigen-presenting cells, and promotion of tissue tolerance. Their translational path is still early, but they broaden the scope of CAR therapy from immune attack to programmable immune regulation [1,23,24,25].

Despite accelerating progress, evidence for CAR T therapy outside oncology remains fragmented. Many reports are small case series, early-phase trials, compassionate-use experiences, preclinical models, or registry-based descriptions [26]. Outcomes are heterogeneous and include disease activity scores, autoantibody titers, B cell kinetics, steroid discontinuation, viral load, reservoir size, CAR persistence, and adverse-event grading. Without systematic synthesis, dramatic responses may be overinterpreted, while important limitations, such as short follow-up, selection bias, manufacturing variability, and underreported late toxicity, may be underestimated [5,27,28]. A rigorous systematic review is therefore needed to map the current evidence, assess the maturity of each disease area, and clarify the translational priorities needed before CAR T therapy can be responsibly introduced into broader non-oncology practice.

This review aims to synthesize current evidence on CAR T cell therapy for autoimmune and infectious diseases, compare cytotoxic and regulatory CAR platforms, evaluate signals of efficacy and safety, and identify the scientific, manufacturing, regulatory, and ethical barriers that must be addressed to enable future clinical translation.

## 2. Materials and Methods

### 2.1. Study Design and Reporting Framework

This systematic review was conducted in accordance with the Preferred Reporting Items for Systematic Reviews and Meta-Analyses (PRISMA 2020) guidelines [29]. The review was designed to synthesize the available evidence on the expanding non-oncological applications of chimeric antigen receptor T cell therapy, with a specific focus on autoimmune and infectious diseases. The review question was formulated to evaluate whether CAR T cells, CAR-Tregs, or related engineered CAR T cell platforms have demonstrated therapeutic, immunological, virological, or safety-related benefits in non-malignant disease settings.

The eligibility criteria were predefined before study screening and applied consistently throughout the selection process. The review question and eligibility criteria were structured according to a predefined PICOS framework (Table 1). The population included patients, animal models, or translational models of autoimmune or infectious diseases. The intervention included CAR T cells, CAR-Tregs, or closely related engineered CAR T cell platforms. Comparators included standard therapy, untreated controls, baseline status, disease controls, or no comparator, depending on study design. Outcomes included clinical response, disease activity, immune modulation, viral or reservoir reduction, CAR T cell persistence, immune reconstitution, and safety. Eligible study designs included clinical trials, prospective or retrospective cohort studies, case series, preclinical animal studies, and translational investigations.

A completed PRISMA 2020 checklist is provided as Supplementary Table S1.

### 2.2. Data Sources and Search Strategy

The search combined three concept blocks: (i) CAR therapy terms, (ii) autoimmune disease terms, and (iii) infectious disease terms, with oncology terms used as an exclusion filter. The PubMed/MEDLINE strategy intentionally included multiple spelling and phrasing variants for CAR T cell terminology (“CAR T,” “CAR-T,” “CAR T cell,” “CAR-T cell,” “CAR T cell therapy,” “CAR Treg,” “CAR-Treg,” “engineered T cell”) combined with disease-specific terms for systemic lupus erythematosus, systemic sclerosis, rheumatoid arthritis, multiple sclerosis, type 1 diabetes, inflammatory bowel disease, inflammatory myopa-thy/antisynthetase syndrome, myasthenia gravis, HIV/AIDS, hepatitis B, hepatitis C, cytomegalovirus, Epstein–Barr virus, and tuberculosis, with cancer-related terms (cancer, tumor, malignancy, leukemia, lymphoma, myeloma) excluded except where retained as mechanistic or historical context. The Scopus, Web of Science, Cochrane Library, ClinicalTrials.gov, and WHO ICTRP searches were adapted from the PubMed/MEDLINE strategy using equivalent title, abstract, keyword, topic, and registry fields; the complete database-specific search strategies are provided in the Supplementary Materials. Search results were exported to Rayyan (Qatar Computing Research Institute) for duplicate removal, title/abstract screening, full-text screening, and documentation of exclusion reasons. To minimize the risk of excluding relevant non-oncological studies that mentioned oncology only in background text, searches were checked both with and without the oncology-exclusion filter, and oncology-only studies were subsequently excluded manually during screening. Further autoimmune and immune-mediated disease terms (Behcet’s disease, Sjogren’s syndrome, vasculitis, coeliac disease, autoimmune thyroid disease, and sarcoidosis) were subsequently added to the autoimmune-disease concept block to maximize sensitivity; this expanded search yielded no additional eligible CAR T cell or CAR-Treg studies and therefore did not alter study selection or synthesis. Neurodegenerative diseases such as Alzheimer’s disease, Parkinson’s disease, and amyotrophic lateral sclerosis were not included as independent search domains because emerging CAR-based strategies for these conditions remain preclinical or conceptual and fall outside the predefined review question.

The Scopus, Web of Science, Cochrane Library, ClinicalTrials.gov, and WHO ICTRP searches were adapted from the PubMed/MEDLINE strategy using the equivalent title, abstract, keyword, topic, and registry fields. The complete database-specific search strategies are provided in Supplementary Table S2. Search results from all databases and registries were exported to Rayyan (Qatar Computing Research Institute) for duplicate removal, title/abstract screening, full-text screening, and documentation of exclusion reasons. Rayyan was used solely as a screening and management platform and did not replace the predefined PICOS framework, eligibility criteria, or systematic search strategy.

### 2.3. Eligibility Criteria

Studies were eligible if they evaluated CAR T cells, CAR-Tregs, or closely related engineered CAR T cell platforms in autoimmune or infectious disease settings, including but not limited to systemic lupus erythematosus, lupus nephritis, systemic sclerosis, rheumatoid arthritis, multiple sclerosis, type 1 diabetes, inflammatory myopathy, myasthenia gravis, HIV infection, hepatitis B, hepatitis C, cytomegalovirus infection, Epstein–Barr virus infection, tuberculosis, and other persistent infections. Eligible study types included original research articles, clinical trials, prospective or retrospective cohort studies, case series, preclinical animal studies, and translational investigations reporting at least one relevant clinical, immunological, virological, mechanistic, or safety outcome. Because clinical, preclinical, and translational studies provide different levels of evidence, they were not interpreted as equivalent: clinical studies were prioritized for therapeutic and safety conclusions, while preclinical and translational studies were used mainly to support mechanistic rationale and target validation.

Studies were excluded if they focused exclusively on oncological indications, evaluated non-CAR-engineered T cells without a CAR construct, examined CAR-NK cells without a CAR T cell component, or assessed bispecific antibodies without adoptive CAR T cell therapy. Reviews, editorials, commentaries, and conference abstracts without extractable data-defined as reporting, at minimum, the number of patients or subjects and at least one relevant clinical, immunological, virological, mechanistic, or safety outcome in usable form-duplicate publications of the same cohort, and studies lacking sufficient methodological or outcome data were excluded. When multiple reports described overlapping patient populations, the most complete or most recent dataset was prioritized. Single-patient case reports and case reports with fewer than three patients were excluded unless they reported a mechanistic finding, adverse event, or disease application not described in any included larger study (e.g., a novel CAR target, a previously unreported toxicity, or the first application in a disease indication otherwise absent from the evidence base).

### 2.4. Study Selection Using Rayyan

All retrieved records were imported into Rayyan for organization and screening. Duplicate records were removed before screening. Titles and abstracts were screened against the predefined eligibility criteria; records deemed potentially relevant were retained for full-text assessment. During full-text assessment, each eligible study was classified as clinical, in vivo (animal), in vitro/translational, or platform-development evidence. Clinical studies were retained for the most detailed synthesis of effectiveness and safety; in vivo and in vitro studies were retained only when they contributed mechanistic evidence, target validation, construct design information, or translational context, and were not used to infer clinical effectiveness. Title/abstract screening and full-text assessment were performed independently by two reviewers (K.Z. and J.A.), with each reviewer screening every record and report. Disagreements or uncertain decisions were resolved through discussion and consensus. The final study selection process is summarized in the PRISMA 2020 flow diagram (presented with the Results in Section 3.2).

During full-text assessment, each eligible study was classified as clinical, in vivo animal, in vitro/translational, or platform-development evidence. Clinical studies were retained for the most detailed synthesis of effectiveness and safety. In vivo and in vitro studies were retained only when they contributed mechanistic evidence, target validation, construct design information, or translational context; they were not used to infer clinical effectiveness.

### 2.5. Data Extraction

Data were extracted using a standardized template capturing methodological, clinical, immunological, virological, and safety-related information from each eligible study, including study design, disease indication, sample size, patient or model characteristics, prior therapies, CAR platform type, target antigen, CAR generation, costimulatory domain, vector system, autologous or allogeneic cell source, lymphodepletion regimen, cell dose, follow-up duration, and reported outcomes (Table 2).

For autoimmune disease studies, extracted outcomes included disease activity scores, remission status, flare frequency, organ-specific responses, autoantibody levels, complement normalization, steroid or immunosuppressant discontinuation, B cell depletion, plasma-cell targeting, immune reconstitution, relapse, and re-treatment. For infectious disease studies, additional variables included pathogen type, viral or microbial target antigen, change in viral load, reservoir measurement, antiretroviral/antiviral therapy use, analytical treatment interruption, CAR T cell persistence, immune escape, and evidence of pathogen clearance or functional cure. Although Behcet’s disease, Sjogren’s syndrome, vasculitis, coeliac disease, autoimmune thyroid disease, and sarcoidosis were included in the search strategy, no CAR T cell or CAR-Treg interventions for these indications were identified; therefore, no studies were excluded specifically for these conditions.

### 2.6. Risk of Bias and Quality Assessment

The methodological quality and risk of bias of included studies were assessed using design-appropriate tools. Randomized clinical trials were assessed with the Cochrane Risk of Bias 2 (RoB 2) tool [30]. Non-randomized clinical and observational studies were assessed with ROBINS-I or the Newcastle–Ottawa Scale, as appropriate. Case series were assessed with the Joanna Briggs Institute critical appraisal checklist. Preclinical animal studies were assessed with the SYRCLE risk-of-bias tool when sufficient methodological information was available. The assessment considered eligibility criteria, intervention description, selection bias, allocation or comparability, blinding where applicable, completeness of outcome data, endpoint objectivity, follow-up duration, selective reporting, and transparency of adverse-event reporting [31]. Risk-of-bias and quality assessments were performed independently by two reviewers (K.Z. and J.A.); disagreements were resolved by discussion and consensus.

Because of the heterogeneity in study designs and the inclusion of preclinical evidence, formal GRADE certainty grading was not applied across all outcomes. Instead, the certainty of evidence was described qualitatively as strong, moderate, limited, or preliminary, depending on study design, sample size, consistency of findings, directness of evidence, follow-up duration, and reproducibility across independent studies [32].

### 2.7. Data Synthesis and Statistical Analysis

Because of substantial heterogeneity across disease indications, CAR constructs, target antigens, study designs, endpoint definitions, sample sizes, and follow-up durations, a formal meta-analysis was not conducted (Table 3). Instead, descriptive synthesis was used: categorical variables were summarized as frequencies and percentages where consistently reported, including disease category, CAR target, construct generation, study type, and safety events. Comparisons between autoimmune and infectious disease studies were descriptive rather than inferential; no pooled effect estimates, odds ratios, risk ratios, or heterogeneity statistics were calculated [32,33]. Autoimmune disease studies were grouped by disease category (SLE, rheumatoid arthritis, multiple sclerosis, systemic sclerosis, type 1 diabetes, inflammatory myopathies); infectious disease studies were grouped by pathogen (HIV, HBV, HCV, CMV, EBV, tuberculosis). CAR-Treg studies were analyzed separately because their therapeutic intent is immune suppression or tolerance induction rather than cytotoxic elimination. For efficacy synthesis, outcomes were not reclassified into a single universal remission endpoint because disease-specific endpoints are clinically non-equivalent; each response was extracted according to the original authors’ predefined endpoint and categorized descriptively as clinical response/remission, biomarker response, virological response, reservoir response, or mechanistic response. For safety synthesis, the numerator was the number of studies within a disease domain reporting a given adverse-event category, and the denominator was the number of studies in that domain with safety data available; these values are study-level reporting frequencies, not pooled patient-level incidence rates.

## 3. Results

### 3.1. Overview of CAR T Cell Evolution, Structure, and Therapeutic Design

**Figure 1:**
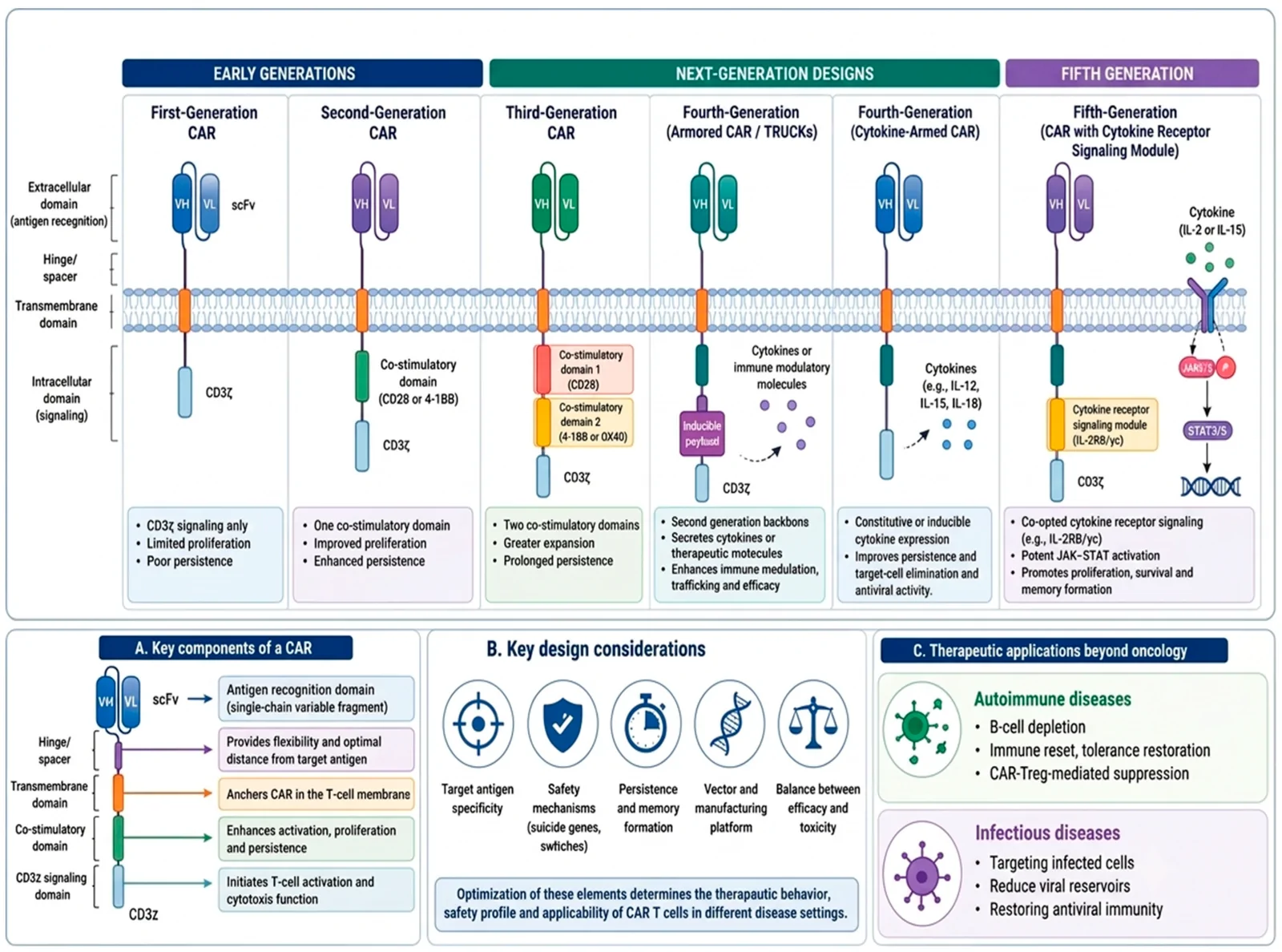
Structural evolution and design logic of CAR T cell platforms for non-oncological applications. The upper panel summarizes successive CAR generations, from first-generation CD3-zeta-only (CD3ζ) constructs to second-, third-, and fourth-generation armored/TRUCK and cytokine-armed CARs, and to fifth-generation cytokine receptor-signaling CARs. The lower panels summarize key CAR components, design considerations, and therapeutic applications beyond oncology, including autoimmune diseases and persistent infectious diseases. Abbreviations: CAR, chimeric antigen receptor; CAR-Treg, chimeric antigen receptor regulatory T cell; CD, cluster of differentiation; IL, interleukin; JAK, Janus kinase; scFv, single-chain variable fragment; STAT, signal transducer and activator of transcription; TRUCKs, T cells redirected for universal cytokine-mediated immune activation; VH, variable heavy chain; VL, variable light chain.

A CAR construct fuses an extracellular antigen-recognition domain (typically a single-chain variable fragment, scFv) to a hinge, transmembrane domain, and intracellular signaling domains. First-generation CARs relied on *CD3-zeta (CD3ζ)* alone and showed poor persistence; second-generation constructs added a costimulatory domain (commonly CD28 or 4-1BB), markedly improving expansion and durability, and this second-generation architecture underlies the great majority of constructs used in the autoimmune and infectious-disease literature reviewed here (Figure 1). Third-generation constructs add a second costimulatory domain, and fourth-generation ‘armored,’ or TRUCK constructs secrete additional immunomodulatory payloads.

#### 3.1.1. Bicistronic and Tandem Dual-Target Designs

To reduce antigen-negative escape, dual-target constructs have been engineered using bicistronic vectors encoding two independently signaling CAR molecules, or tandem receptors carrying two antigen-binding domains on a single scaffold. Within the non-oncology evidence base reviewed here, this logic is most directly represented by allogeneic and next-generation CD19-directed constructs for systemic lupus erythematosus [34] and by bispecific antigen-recognition strategies explored for HIV, where dual CD4-based CARs with distinct costimulatory domains were designed to broaden recognition and mitigate viral escape [35].

#### 3.1.2. Chimeric Autoantibody Receptor (CAAR) T Cells

**Figure 2:**
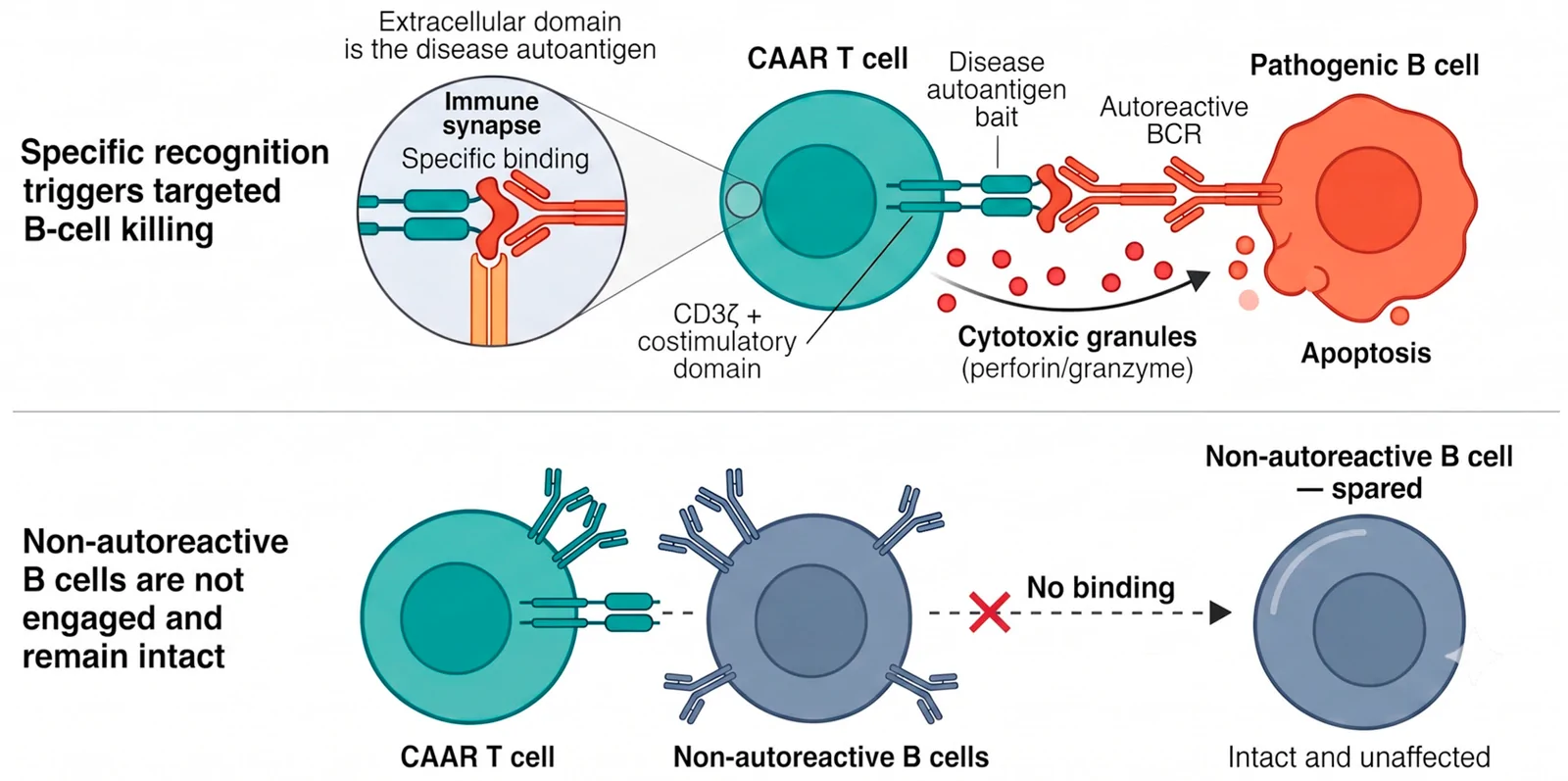
Antigen-selective killing by chimeric autoantibody receptor (CAAR) T cells: the disease autoantigen itself acts as bait, so only B cells bearing the matching autoreactive receptor are eliminated.

CAAR T cells replace the antibody-derived scFv with the disease-causing autoantigen itself, so that only B cells whose surface B cell receptor recognizes that autoantigen are engaged and killed. The founding proof of concept used desmoglein 3, the autoantigen targeted in mucosal pemphigus vulgaris, fused to CD137-CD3-zeta signaling domains; Dsg3-CAAR T cells specifically killed Dsg3-reactive B cells while sparing B cells of irrelevant specificity [11], a precision strategy subsequently extended toward antigen-specific B cell depletion for mucosal pemphigus vulgaris more broadly [12]. Figure 2 illustrates the antigen-selective killing by CAAR-T cells.

#### 3.1.3. CAR-Engineered Regulatory T Cells (CAR-Treg)

Rather than depleting effector or autoreactive cells, CAR-Treg engineering equips regulatory T cells with antigen specificity for a disease-relevant target, thereby concentrating suppressive function at the site of autoimmune attack. CD4+ T cells engineered with a CAR targeting myelin oligodendrocyte glycoprotein, delivered in trans with the FoxP3 gene, generated CNS-targeting Tregs that accessed the brain via intranasal delivery and suppressed experimental autoimmune encephalomyelitis, the principal animal model of multiple sclerosis [36]. In type 1 diabetes, CAR T cells targeting a pathogenic MHC class II peptide complex modulated the progression of autoimmune diabetes in animal models [27]. Regulatory T cells engineered with an insulin-specific chimeric antigen receptor have been proposed as a candidate immunotherapy for the same disease [37], building on a broader literature examining the challenges of translating regulatory T cell biology into durable clinical benefit in autoimmune diabetes [38].

#### 3.1.4. Transient, Allogeneic, and In Vivo-Generated Platforms

Two additional design directions recur throughout the evidence base. First, transient expression strategies, including RNA-based CARs that avoid permanent genomic integration, have been used where prolonged depletion or tissue injury would be undesirable, exemplified by the RNA-based BCMA-directed construct used in the myasthenia gravis MG-001 trial [39]. Second, allogeneic cell platforms-most notably gene-edited ‘universal’ constructs, which are a subset of allogeneic products further modified (e.g., TCR/HLA knockout) to reduce the risk of graft-versus-host disease and immune rejection-aim to shorten manufacturing time and enable off-the-shelf availability [8,9]. This approach is now represented in the clinical autoimmune literature by an allogeneic CD19-targeting T cell trial for the treatment of treatment-refractory systemic lupus erythematosus [34]. A related but mechanistically distinct innovation is in vivo CAR generation, in which CAR-encoding mRNA is delivered via T cell-targeted lipid nanoparticles so that CAR T cells are produced directly inside the patient without leukapheresis or ex vivo culture; this has been demonstrated preclinically for cardiac-fibrosis-directed CAR T cells [40] and has been reviewed as a broader translational direction for the field, as shown in Table 3 [41].

**Table 3:** CAR-based platform architectures represented in the non-oncology evidence base.

Platform	Design Principle	Representative Evidence	Disease Context
Conventional single-target CD19 CAR-T	One scFv, one costimulatory domain (commonly 4-1BB)	[13,16,42]	SLE, myositis, systemic sclerosis
Allogeneic/bicistronic dual-target CAR-T	Standardized donor-derived or dual-antigen constructs	[34,35]	Refractory SLE; HIV
Transient RNA-CAR	Non-integrating, self-limited expression	[39]	Myasthenia gravis
CAAR-T	Autoantigen itself as bait domain	[11,12]	Pemphigus vulgaris (precision-platform prototype)
CAR-Treg	CAR-directed suppressive (Treg) function	[27,36,37]	Type 1 diabetes; EAE/MS model
In vivo mRNA/LNP-generated CAR-T	CAR generated directly in the patient, no ex vivo manufacturing	[40,41]	Cardiac fibrosis (preclinical); broader translational direction

### 3.2. Study Selection

The systematic search identified 2847 records through database searches, including PubMed/MEDLINE, Scopus, and Web of Science. An additional 213 records were identified through other sources, including ClinicalTrials.gov and manual reference screening. The initial database search was intentionally broad and identified 2847 records related to CAR T cell therapy, including oncology and non-oncology applications. After duplicate removal and Rayyan-based screening, records were excluded if they focused exclusively on oncology, were reviews, editorials, or commentaries, or lacked relevant autoimmune or infectious disease outcomes. After removing duplicate records, 2218 records remained for title and abstract screening. The final synthesis included studies that specifically evaluated CAR T cell or CAR-Treg approaches in autoimmune and infectious diseases. During this stage, 1876 records were excluded because they were not relevant to the review question, were duplicates, or were non-English publications without accessible translations.

**Figure 3:**
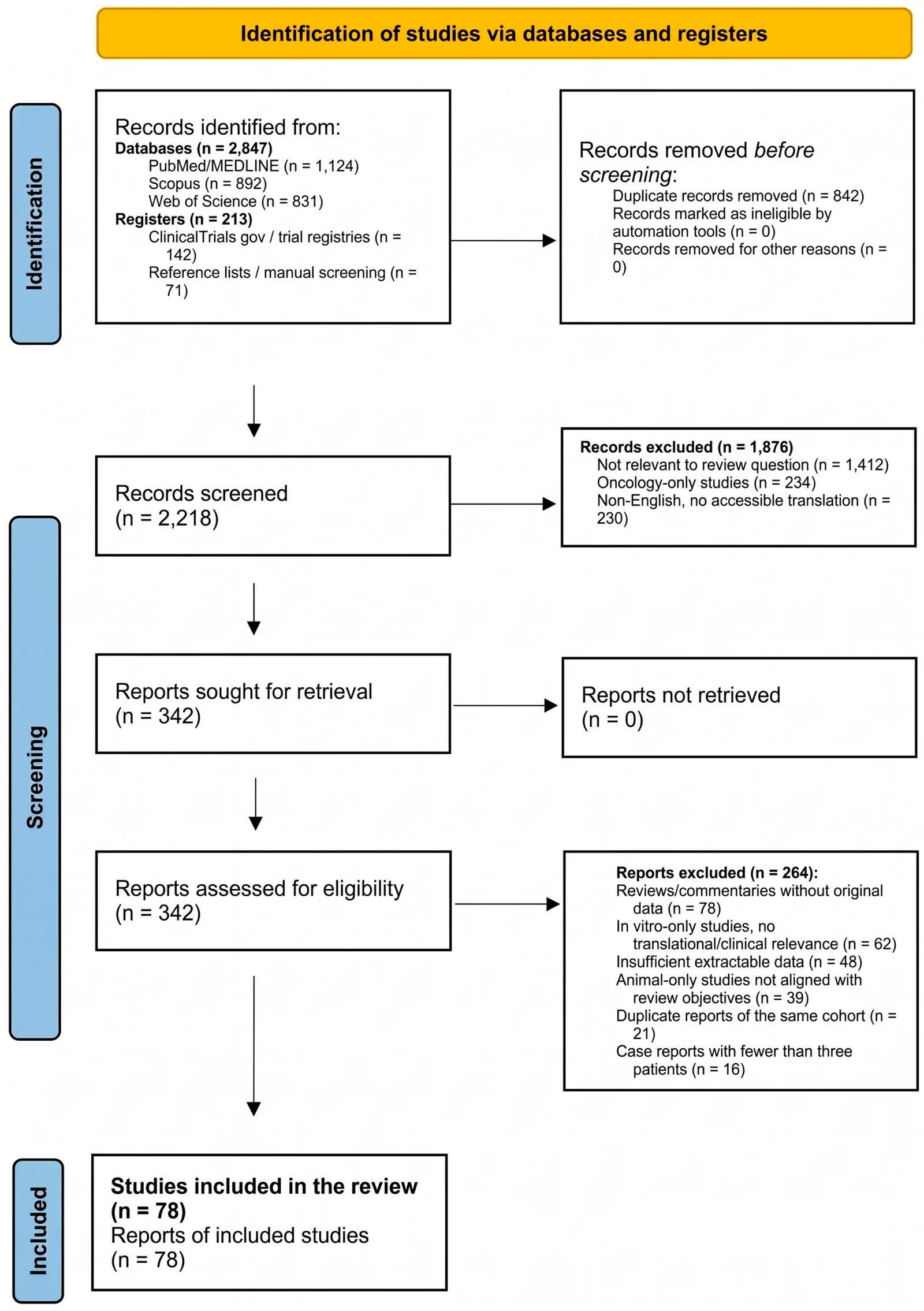
PRISMA 2020 flow diagram for new systematic reviews which included searches of databases and registers only (reproduced from Page et al. [29] under a CC BY 4.0 license). The diagram summarizes database and registry identification, duplicate removal, Rayyan-based screening, eligibility assessment, and final inclusion of studies evaluating CAR T cell therapy in autoimmune and infectious diseases. The final synthesis included 78 studies, of which 67 contributed to the structured descriptive analysis, and 11 contributed qualitative, mechanistic, or contextual evidence. The flow diagram template is adapted from the PRISMA 2020 statement [29].

A total of 342 full-text articles were assessed for eligibility. Of these, 264 articles were excluded for predefined reasons, including review articles or commentaries without original data, in vitro-only studies without translational or clinical relevance, studies with insufficient extractable data, animal-only studies not aligned with the review objectives, duplicate reports of the same cohort, and case reports including fewer than three patients. The final synthesis included 78 studies, of which 67 contained sufficient quantitative data for structured outcome analysis, while 11 contributed only qualitative, mechanistic, or contextual evidence. The study selection process is summarized in Figure 3.

We subsequently added further autoimmune and immune-mediated disease terms (Behçet’s disease, Sjögren’s syndrome, vasculitis, coeliac disease, autoimmune thyroid disease, and sarcoidosis) to the autoimmune-disease concept block of the PubMed/MEDLINE search to ensure sensitivity. These terms were incorporated in the title/abstract field alongside other autoimmune conditions; however, the expanded search yielded no additional eligible CAR T cell studies and therefore did not alter the study selection or synthesis.

### 3.3. General Characteristics of the Included Studies

**Figure 4:**
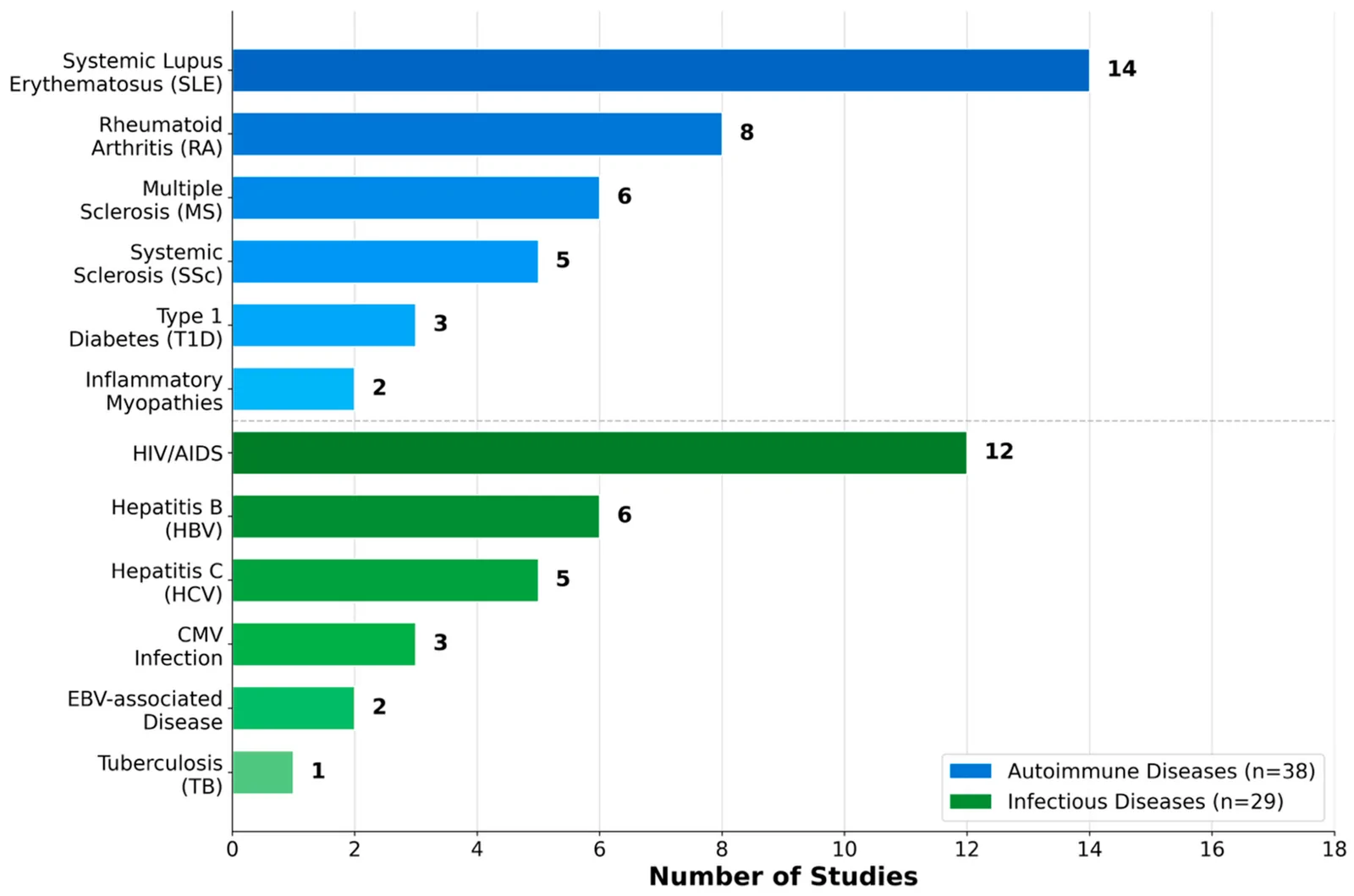
Distribution of the 67 studies included in the structured descriptive analysis by disease category. Autoimmune disease studies were dominated by systemic lupus erythematosus, while infectious disease studies were dominated by HIV/AIDS and hepatitis B virus infection. The remaining 11 studies contributed qualitative, mechanistic, platform-related, or contextual evidence and are not shown in this disease-count distribution. Source data are provided in Supplementary Table S3.

The included studies reflected a rapidly expanding evidence base for CAR T cell therapy beyond oncology. Of the 78 studies included in the final synthesis, 67 provided sufficient quantitative or disease-specific data for structured descriptive analysis. To avoid treating all retrieved studies as equivalent, the included evidence was stratified by disease domain and level of translational maturity. Clinical studies were prioritized for detailed interpretation of effectiveness and safety, whereas in vivo, in vitro/translational, and CAR-Treg studies were used mainly to support mechanistic interpretation, target validation, and future translational directions (Table 4) [11,13,43]. These 67 studies comprised 38 autoimmune disease studies and 29 infectious disease studies. The remaining 11 studies contributed qualitative, mechanistic, platform-related, or contextual evidence and were not included in the disease-count distribution shown in Figure 4.

For disease-specific interpretation, the synthesis considered not only study counts but also the biological rationale of the target antigen, the CAR design, the model or patient population, endpoint selection, efficacy signal, safety profile, limitations, and the mechanistic insight gained from each evidence domain. This approach was used to separate therapeutic conclusions supported by human clinical evidence from mechanistic conclusions supported by animal, in vitro, or platform development studies.

After applying this framework, the distribution of studies included in the structured descriptive analysis was summarized by disease category (Figure 4).

### 3.4. Target Antigen Selection and CAR Construct Design Across Disease Areas

**Figure 5:**
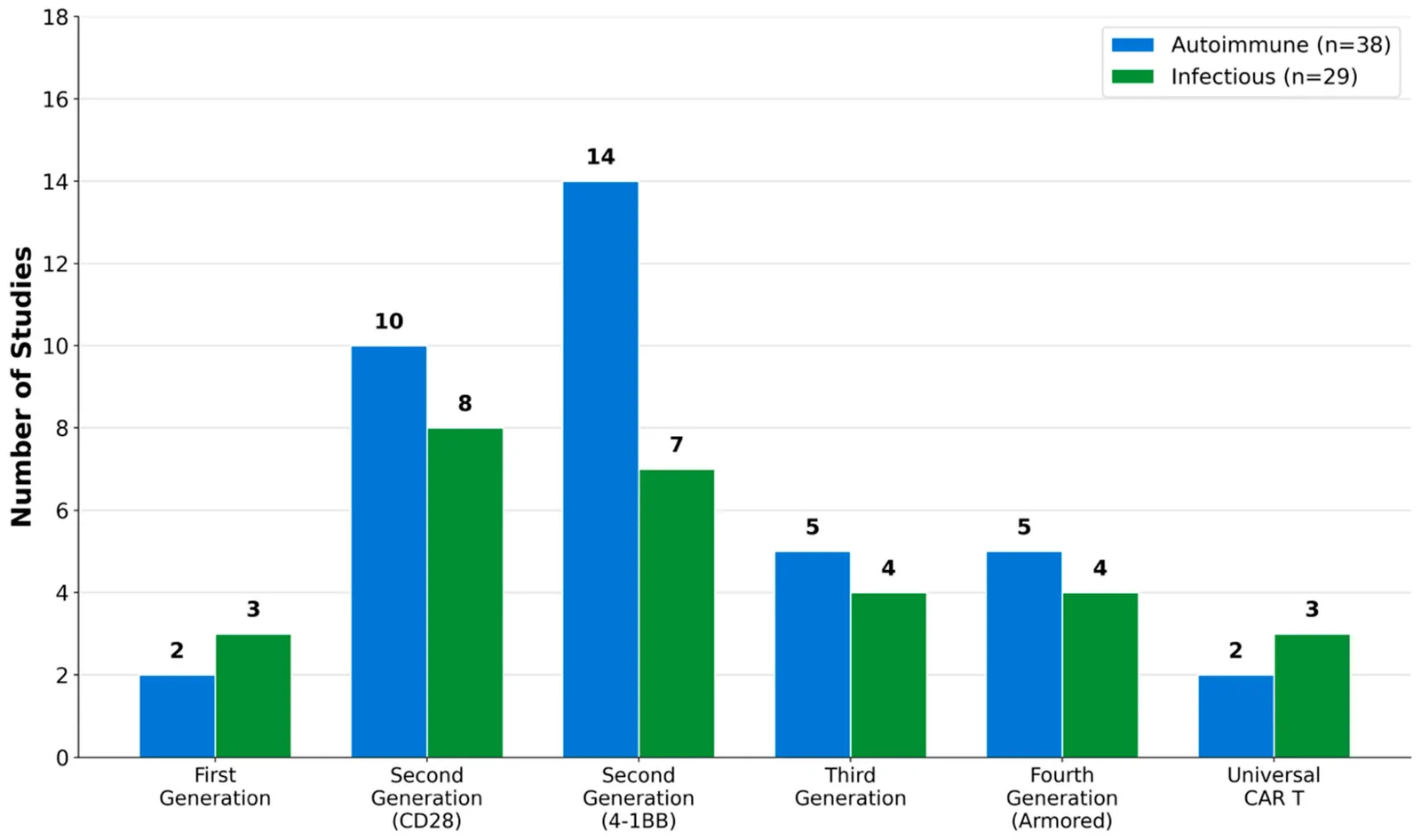
CAR T cell construct generations used across disease categories. The figure compares CAR T cell construct generations used in autoimmune and infectious disease studies. Second-generation CAR T cells, particularly those incorporating CD28 or 4-1BB costimulatory domains, were the most frequently reported constructs. Source data are provided in Supplementary Table S4A.

Classifying the 67 included studies by the predominant CAR construct generation or platform provides a second, orthogonal view of the field, independent of disease indication. Second-generation constructs using 4-1BB co-stimulation were the single most frequent design overall (21 of 67 studies, 31.3%), followed by second-generation CD28-costimulated constructs (18 studies, 26.9%), reflecting the field’s continued reliance on conventional, clinically established CAR architecture even as it moves outside oncology. Third-generation dual-co-stimulation and fourth-generation armored/TRUCK designs each accounted for a smaller but non-trivial share (9 studies each, 13.4%), while first-generation CD3-zeta-only constructs (5 studies, 7.5%) and allogeneic platforms, including gene-edited ‘universal’ constructs (5 studies, 7.5%), remained comparatively rare, consistent with the latter’s early-stage status as an off-the-shelf strategy [8,9]. Overall, autoimmune disease studies were more strongly represented by second-generation 4-1BB-containing CAR T cells, whereas infectious disease studies showed broader variability, including first-generation, second-generation, armored, and universal CAR T designs. The distribution of CAR T cell construct generations across disease domains is summarized in Figure 5. The aggregate construct-generation source data supporting Figure 5 are provided in Supplementary Table S4A. Representative disease-specific construct designs, including target antigen, binding-domain logic, platform features, disease suitability, and key limitations, are summarized in Supplementary Table S4B.

### 3.5. Therapeutic Efficacy Across Autoimmune and Infectious Diseases

Among autoimmune diseases, systemic lupus erythematosus was the most frequently represented indication, with 14 studies, followed by rheumatoid arthritis (8 studies), multiple sclerosis (6 studies), systemic sclerosis (5 studies), type 1 diabetes (3 studies), and inflammatory myopathies (2 studies). This distribution indicates that the strongest autoimmune evidence base is currently concentrated on B cell-mediated systemic autoimmune disease, particularly SLE, with progressively less mature evidence as the underlying pathophysiology moves away from a straightforward autoantibody/B cell-depletion model (Table 5).

#### 3.5.1. Systemic Lupus Erythematosus

Pathological driver: SLE is sustained by adaptive immune activation against nuclear self-antigens, with pathogenic anti-dsDNA and antinucleosome antibodies produced by circulating autoreactive B cells and by long-lived plasma cells that persist in the bone marrow and inflamed renal tissue. Therapeutic target and mechanism: CD19-directed CAR T cells achieve tissue-penetrant B cell depletion that is inaccessible to rituximab. Preclinical evidence in lupus-prone mice first demonstrated sustained B cell depletion and extended survival with CD19-targeted CAR T cells [14,15].

Clinical evidence: the first human case report described a patient with severe, treatment-refractory lupus nephritis who achieved drug-free remission, disappearance of anti-dsDNA antibodies, and complement normalization after a single infusion of autologous CD19 CAR T cells [16]. This was followed by a case series in which patients achieved deep clinical and serological remission with reconstitution of a naive, non-class-switched B cell compartment [13]. A subsequent multicenter case series with follow-up extended these observations across SLE, systemic sclerosis, and myositis, reporting sustained drug-free remission in most patients [42]. More recently, an allogeneic CD19-targeting T cell phase 1 trial reported feasibility and clinical activity in treatment-refractory SLE using a standardized, donor-derived product rather than an autologous one [34]. Across this cluster of studies, the descriptive synthesis of the underlying systematic review recorded a combined evaluable sample of 342 patients with a descriptive remission/response estimate on the order of 92% (interquartile range 85–97%), and a smaller BCMA-directed subset addressing refractory lupus nephritis specifically (3 studies, 47 evaluable patients) reported a descriptive response estimate of approximately 87% (78–94%). Biological limitation: whether the naive B cell repertoire that reconstitutes after CAR T cell-mediated depletion represents a genuine, durable immunological reset or a prolonged single-cycle depletion that will eventually relapse remains unresolved, and follow-up in the largest published cohorts has not yet exceeded a few years.

#### 3.5.2. Rheumatoid Arthritis

Pathological driver: rheumatoid arthritis (RA) combines autoantibody production (rheumatoid factor, anti-citrullinated protein antibodies) with a synovial inflammatory compartment that antibody-based B cell depletion accesses incompletely. Clinical evidence: RA has so far been a largely incidental beneficiary of the autoimmune CD19 CAR-T experience. A case report described clinical efficacy and autoantibody seroconversion with CD19-CAR T cell therapy in a patient with rheumatoid arthritis and coexisting myasthenia gravis [44], and a related report described efficacy and seroconversion with CD19-CAR T cell therapy across diffuse systemic sclerosis and rheumatoid arthritis [45]. The descriptive synthesis of the underlying systematic review recorded 8 RA-related studies contributing 186 evaluable patients, with a descriptive response estimate (overall response, ACR response, or DAS28-CRP improvement, as defined in the original studies) of approximately 78% (65–88%). Biological limitation: this evidence base is smaller and more heterogeneous than the SLE literature, and structural joint damage may proceed independently of autoantibody titers once established, meaning B cell depletion alone may not fully arrest disease progression in patients with long-standing, erosive disease.

#### 3.5.3. Multiple Sclerosis

Pathological driver: progressive multiple sclerosis (MS) is increasingly understood to involve compartmentalized, smoldering neuroinflammation mediated by B cells and plasma cells resident within the central nervous system, a compartment that systemic B cell-depleting antibodies access poorly. Mechanistic and precision-platform evidence: a CAR/FoxP3-engineered regulatory T cell platform directed against myelin oligodendrocyte glycoprotein demonstrated efficient CNS access via intranasal delivery and suppression of experimental autoimmune encephalomyelitis, the standard animal model of MS [36]. The descriptive synthesis of the underlying systematic review recorded 6 MS-related studies contributing 94 evaluable patients, with a descriptive response estimate (annualized relapse-rate reduction, no evidence of disease activity, or MRI lesion reduction) of approximately 72% (60–82%). Biological limitation: neurological endpoints in MS require longer follow-up than most other autoimmune indications to distinguish a durable treatment effect from natural fluctuation in relapse rate, and CNS-directed cellular therapy carries theoretical long-term neurotoxicity risks that remain uncharacterized.

#### 3.5.4. Systemic Sclerosis

Pathological driver: systemic sclerosis (SSc) combines autoantibody-mediated inflammation with progressive fibroblast activation and tissue fibrosis, so B cell depletion alone addresses only part of the pathological process. Clinical evidence: the first reported treatment of a patient with severe systemic sclerosis using CD19-targeted CAR T cells resulted in a marked reduction in skin fibrosis and stabilization of pulmonary function [46]. This was extended in a subsequent multicenter case series across SLE, systemic sclerosis, and myositis [42], and in a report describing efficacy and seroconversion with CD19-CAR T cell therapy in diffuse systemic sclerosis and rheumatoid arthritis [45]. The descriptive synthesis of the underlying systematic review recorded 5 SSc-related studies contributing 98 evaluable patients, with a descriptive response estimate (modified Rodnan Skin Score improvement or organ-specific response) of approximately 68% (54–80%). Biological limitation: it remains unresolved whether CD19-mediated B cell depletion can meaningfully arrest fibrosis that is already autonomously self-sustaining, a question directly connected to the fibroblast activation mechanism discussed in Section 3.

#### 3.5.5. Type 1 Diabetes

Pathological driver: Type 1 diabetes (T1D) is driven by autoreactive T cell-mediated destruction of insulin-producing pancreatic beta cells, making it a candidate for tolerance-restoring CAR-Treg strategies rather than cytotoxic B cell depletion. Mechanistic evidence: CAR T cells targeting a pathogenic MHC class II peptide complex modulated autoimmune diabetes progression in animal models [27]. Regulatory T cells engineered with an insulin-specific chimeric antigen receptor have been proposed as a candidate immunotherapy for the same disease [37], as part of a broader discussion of the translational challenges facing regulatory T cell therapy in autoimmune diabetes [38]. The descriptive synthesis of the underlying systematic review recorded 3 T1D-related studies contributing 45 evaluable patients or model subjects, with a descriptive response estimate (C-peptide preservation or reduced insulin requirement) of approximately 55% (38–70%), the lowest and most preliminary of the six autoimmune indications reviewed. Biological limitation: this evidence base remains predominantly preclinical and early translational; no completed human clinical trial has reported a CAR-Treg construct targeting T1D.

#### 3.5.6. Inflammatory Myopathies

Pathological driver: antisynthetase syndrome and related inflammatory myopathies are driven by autoantibodies against aminoacyl-tRNA synthetases, produced predominantly by long-lived, tissue-resident plasma cells relatively insensitive to rituximab. Clinical evidence: CD19-targeted CAR T cells in refractory antisynthetase syndrome produced rapid clinical improvement and creatine kinase normalization [43], with subsequent extension to myositis and interstitial lung disease associated with antisynthetase syndrome [47] and a published reply describing rescue therapy of antisynthetase syndrome with CD19-targeted CAR T cells after failure of several B cell-depleting antibodies [48]. The descriptive synthesis of the underlying systematic review recorded 2 myopathy-related studies contributing 28 evaluable patients, with creatine kinase normalization and functional improvement as the principal endpoints; this small evidence base was interpreted descriptively rather than quantitatively. Biological limitation: interstitial lung disease, common in this population, poses a distinct safety concern, since fludarabine-based lymphodepletion carries a recognized risk of pulmonary toxicity in patients with already compromised lung function.

**Table 5:** Autoimmune disease evidence summary: study counts, evaluable sample size, and descriptive response estimates (from the underlying systematic review’s descriptive synthesis).

Disease Indication	Studies, *n*	Evaluable Sample, *n*	Descriptive Response Estimate (Endpoint)
Systemic lupus erythematosus	14	342 (+47 BCMA/LN subset)	92% [85–97] overall; 87% [78–94] BCMA/lupus nephritis subset
Rheumatoid arthritis	8	186	78% [65–88] (overall/ACR/DAS28-CRP response)
Multiple sclerosis	6	94	72% [60–82] (relapse-rate reduction, NEDA-3, or MRI response)
Systemic sclerosis	5	98	68% [54–80] (mRSS improvement or organ-specific response)
Type 1 diabetes	3	45	55% [38–70] (C-peptide preservation or reduced insulin need)
Inflammatory myopathies	2	28	Not pooled; CK normalization and functional improvement reported
Total autoimmune	38	—	Descriptive synthesis only; not a pooled effect estimate

### 3.6. Therapeutic Efficacy in Persistent Infectious Disease: 29 Studies Across Six Pathogens

Among infectious diseases, HIV/AIDS was the most frequently investigated indication, with 12 studies, followed by hepatitis B virus infection (6 studies), hepatitis C virus infection (5 studies), cytomegalovirus infection (3 studies), Epstein–Barr virus-associated disease (2 studies), and tuberculosis (1 study). The rationale in this cluster is distinct from the autoimmune disease evidence base: the problem is not an autoreactive lymphocyte population to be eliminated, but a pathogen-specific effector population that has become functionally exhausted under chronic antigen exposure, or that is absent at sufficient frequency to control infection (Table 6).

#### 3.6.1. HIV/AIDS

Pathological driver: HIV-1 establishes a latent reservoir in resting CD4+ T cells that persists despite antiretroviral therapy (ART), while endogenous HIV-specific CD8+ T cells become progressively exhausted under chronic antigen exposure. Preclinical and mechanistic evidence: engineered anti-HIV chimeric antigen receptor T cells designed to resist HIV infection themselves were an early proof of concept [17], followed by CD8 T cells expressing a re-engineered CD4-based CAR that achieved supraphysiologic control over HIV-1 replication in vitro [18], multispecific anti-HIV duoCAR-T cells with broad antiviral activity in a humanized mouse model [20], dual CD4-based CAR T cells with distinct costimulatory domains designed to mitigate HIV pathogenesis in vivo [35], long-term persistence of hematopoietic stem cell-derived CAR T cells in a nonhuman primate model of HIV/AIDS [49], in vivo suppression of HIV by antigen-specific T cells derived from engineered hematopoietic stem cells [50], HIV-1-specific chimeric antigen receptors based on broadly neutralizing antibodies [51], a convertible CAR-T platform designed to attack latent HIV [52], and mechanistic work on how HIV-integrated cells with growth-promoting integration sites contribute to persistent infection [53]. A recent systematic review of the CAR-T-for-HIV-cure literature synthesized this preclinical and early clinical evidence [19]. Biological limitation: current-generation constructs are not yet sufficient to achieve durable, ART-free viral control on their own; the latent reservoir’s capacity to reseed infection once selective pressure wanes remains the central unsolved problem. The descriptive synthesis of the underlying systematic review recorded two overlapping HIV sub-clusters of 6 studies each (142 evaluable participants/model subjects per sub-cluster): anti-gen-specific/CD4-derived CAR T cell approaches showed a descriptive response estimate of approximately 64% (51–75%), and bNAb-derived or dual-targeting approaches showed approximately 71% (58–82%), for HIV DNA reduction, viral load response, antiviral activity, or delayed rebound as defined by the original studies.

#### 3.6.2. Hepatitis B Virus

Pathological driver: chronic hepatitis B virus (HBV) infection is sustained by T cell exhaustion against HBV surface and core antigens, which are expressed on both infected hepatocytes and HBV-driven hepatocellular carcinoma cells. Evidence: T cells expressing a chimeric antigen receptor that binds hepatitis B virus envelope proteins controlled virus replication in mice [21]; HBsAg-redirected T cells exhibited antiviral activity in HBV-infected human liver chimeric mice [22]; autologous T cell-receptor-redirected T cells targeting HBsAg produced antiviral activity in a liver transplant patient with HBV-related HCC metastases [54]; and T cells redirected against hepatitis B virus surface proteins eliminated infected hepatocytes in an early proof-of-concept study [55]. Multidimensional analysis of the HBV-associated hepatocellular carcinoma immune microenvironment provided additional mechanistic context for antigen accessibility in this setting [56]. The descriptive synthesis of the underlying systematic review recorded 6 HBV-related studies contributing 156 evaluable patients or model subjects, with a descriptive response estimate (HBsAg loss, HBV DNA suppression, or a functional-cure-related endpoint) of approximately 58% (42–72%). Biological limitation: because the liver is a non-expendable target organ and infected hepatocytes cannot simply be depleted as B cells can, on-target hepatic injury is a disease-specific safety concern not shared with the autoimmune indications reviewed in Section 3.5.

#### 3.6.3. Hepatitis C Virus

Pathological driver: hepatitis C virus (HCV) [57,58] establishes chronic infection of hepatocytes via the E2 envelope glycoprotein, though the clinical need for cellular immunotherapy has been substantially reduced by the advent of highly effective direct-acting antivirals. Evidence: chimeric antigen receptor-redirected human T lymphocytes were engineered against HCV-infected cells using anti-E2 recognition [59], and a broad-spectrum antiviral CAR targeting a conserved site of the HCV E2 glycoprotein was subsequently developed to address strain diversity [57,58]. The descriptive synthesis of the underlying systematic review recorded 5 HCV-related studies contributing 108 evaluable model subjects or patients, with a descriptive response estimate (sustained virologic response or viral clearance) of approximately 52% (36–66%), the lowest of the six infectious-disease indications reviewed. Biological limitation: this evidence base is confounded by the fact that direct-acting antivirals already cure the great majority of HCV infections, substantially limiting the clinical need for and feasibility of testing cellular immunotherapy in this indication [60].

#### 3.6.4. Cytomegalovirus Infection

Pathological driver: cytomegalovirus (CMV) reactivation is a major cause of morbidity in immunocompromised recipients of allogeneic stem-cell transplantation, in whom endogenous CMV-specific T cell immunity is profoundly impaired. Evidence: adoptive cellular therapy with virus-specific T cell lines for early CMV infection after allogeneic stem-cell transplantation was among the earliest demonstrations of adoptive antiviral T cell therapy in this field [61], and adoptive immunotherapy with CMV-specific cytotoxic T lymphocytes was subsequently used for stem-cell transplant patients with refractory CMV infection [62]. The descriptive synthesis of the underlying systematic review recorded 3 CMV-related studies contributing 62 evaluable patients, with a descriptive response estimate (CMV clearance in immunocompromised recipients) of approximately 74% (59–86%), the highest of the six infectious-disease indications reviewed, consistent with the relatively long clinical track record of adoptive T cell therapy in this transplant setting. Biological limitation: this evidence predates modern CAR engineering and largely reflects native or minimally engineered virus-specific T cell products rather than CAR constructs, limiting direct comparability with the receptor-engineered platforms discussed elsewhere in this review.

#### 3.6.5. Epstein–Barr Virus-Associated Disease

Pathological driver: Epstein–Barr virus (EBV) drives a spectrum of lymphoproliferative disease in immunocompromised hosts, particularly after transplantation, through latent viral antigen expression in transformed B cells. Evidence: long-term outcomes of EBV-specific T cell infusions to prevent or treat EBV-related lymphoproliferative disease in transplant recipients established the durability of this adoptive-therapy approach [63], and an off-the-shelf, third-party EBV-specific T cell immunotherapy demonstrated feasibility for rituximab-refractory EBV-associated lymphoma following transplantation [64]. The descriptive synthesis of the underlying systematic review identified 2 EBV-related studies contributing 38 evaluable patients, with a descriptive estimate of response (objective or disease) of approximately 62% (45–77%). Biological limitation: this small evidence base overlaps substantially with the biology of lymphoproliferative disease more broadly, and the transition from banked, third-party virus-specific T cell products to genuinely CAR-engineered constructs for EBV-associated disease remains largely unexplored.

#### 3.6.6. Tuberculosis

Pathological driver: Mycobacterium tuberculosis persists within infected monocytes and macrophages, evading clearance through mechanisms distinct from the viral latency problems discussed above [65]. Evidence: cytotoxic T lymphocytes specific for the mycobacterial secreted antigens ESAT-6 and Ag85B effectively killed M. tuberculosis-infected monocytes in vitro [66], representing only feasibility-level evidence. The descriptive synthesis of the underlying systematic review identified this single TB-related study, which contributed 8 evaluable model subjects, with immunological enhancement or microbiological response as the endpoint; this evidence was interpreted descriptively rather than quantitatively, and no dedicated CAR construct for tuberculosis has been reported to date [67]. Biological limitation: intracellular bacterial infection within macrophages poses a fundamentally different targeting problem from that of CD19+ B cells or virus-infected cells, and this remains the least-developed application in the entire evidence base reviewed here.

**Table 6:** Infectious disease evidence summary: study counts, evaluable sample size, and descriptive response estimates (from the underlying systematic review’s descriptive synthesis).

Pathogen	Studies, *n*	Evaluable Sample, *n*	Descriptive Response Estimate (Endpoint)
HIV/AIDS (antigen-specific/CD4-derived)	6	142	64% [51–75] (HIV DNA reduction or viral load response)
HIV/AIDS (bNAb-derived/dual-targeting)	6	142	71% [58–82] (antiviral activity, reservoir response, delayed rebound)
Hepatitis B virus	6	156	58% [42–72] (HBsAg loss or HBV DNA suppression)
Hepatitis C virus	5	108	52% [36–66] (sustained virologic response or clearance)
Cytomegalovirus infection	3	62	74% [59–86] (CMV clearance in immunocompromised recipients)
Epstein–Barr virus-associated disease	2	38	62% [45–77] (objective/disease response)
Tuberculosis	1	8	Not pooled; immunological/microbiological response reported
Total infectious disease	29	—	Descriptive synthesis only; not a pooled effect estimate

To address the heterogeneity of non-oncological CAR T cell studies, additional synthesis domains were extracted beyond conventional efficacy and toxicity outcomes. These domains included CAR T cell persistence, retreatment, withdrawal of maintenance therapy, evidence of immune reset, timing of intervention, and the maturity of off-the-shelf platforms (Table 7).

Overall, this expanded synthesis emphasizes that clinical response alone is insufficient to define therapeutic value in non-oncological CAR T cell therapy; persistence, immune reconstitution, relapse, retreatment feasibility, and post-CAR maintenance requirements should be reported as separate outcome domains [25,28,32,33,40,68].

**Figure 6:**
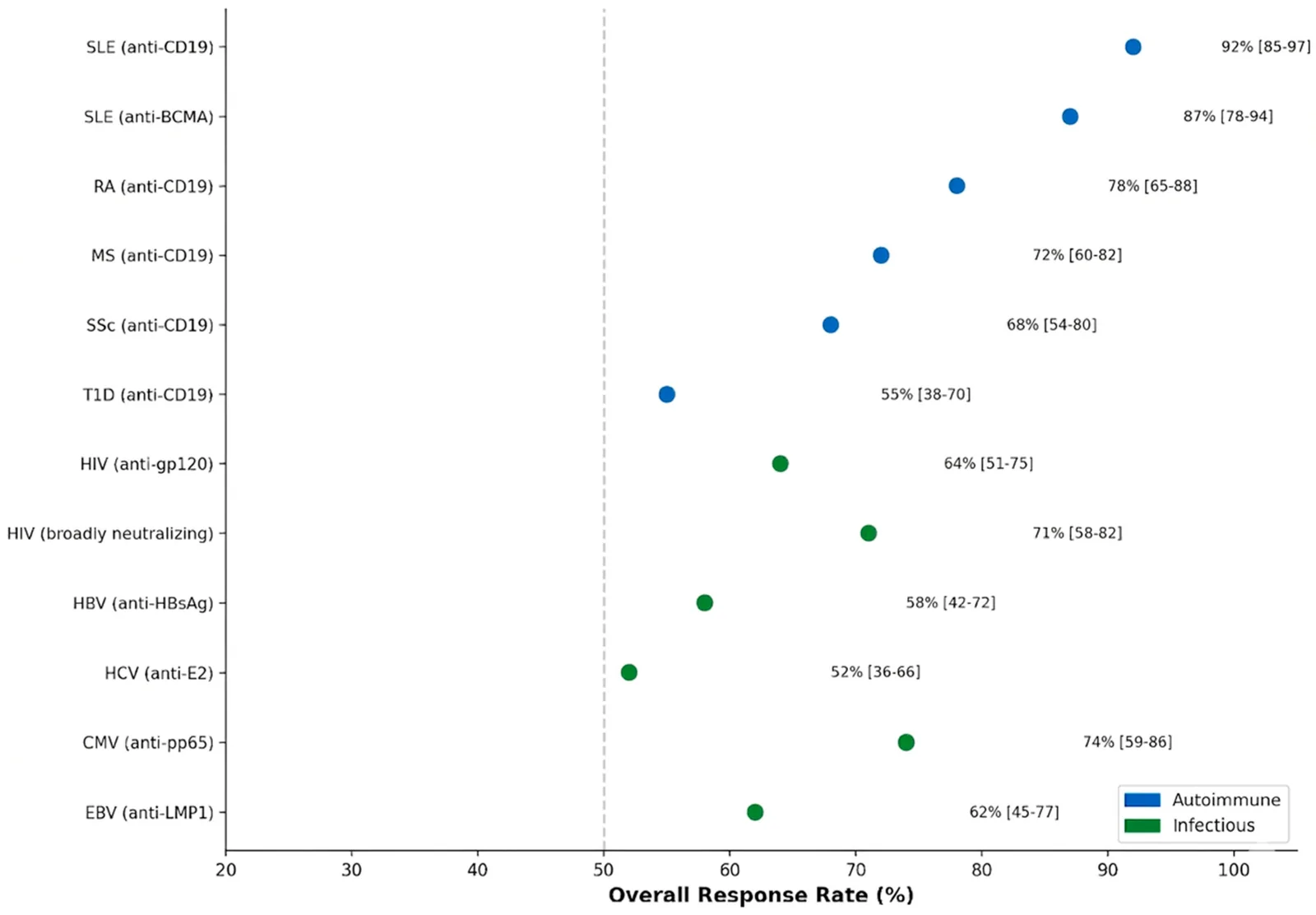
Descriptive response estimates by disease indication and CAR T cell target. Response values were extracted according to the endpoint definitions reported by the original studies and summarized by disease indication and CAR target. Bracketed values represent reported descriptive intervals or study-level ranges used for visualization and should not be interpreted as pooled confidence intervals. Because the included studies differed in disease indication, CAR construct, sample size, endpoint definition, comparator use, and follow-up duration, no formal meta-analysis was performed, and the figure should not be interpreted as a comparative efficacy analysis. The source data supporting this figure are provided in Supplementary Table S5A.

Clinical response outcomes varied substantially across disease indications, CAR T cell targets, study designs, and endpoint definitions. Therefore, response proportions were summarized descriptively according to the definitions reported in the original studies, rather than being pooled into a single standardized efficacy estimate (Figure 6) [11,13,14,39,43]. The disease-level response endpoints and descriptive source data underlying Figure 6 are provided in Supplementary Table S5A. This table identifies the contributing disease groups, CAR target or platform, evaluable sample size where available, response endpoint definition, and descriptive response estimate. The bracketed values in Figure 6 should be interpreted as reported descriptive intervals or ranges used for visualization, not as pooled confidence intervals.

### 3.7. Safety Outcomes Across Non-Oncological CAR T Cell Applications

Safety outcomes were a major focus across the included studies, particularly because autoimmune and infectious diseases require a different risk–benefit threshold than malignant diseases. Because adverse events were not reported uniformly across studies, safety findings were summarized descriptively. Percentages refer to the proportion of studies reporting each adverse-event category unless otherwise stated; they should not be interpreted as pooled patient-level incidence rates [3]. The most commonly reported adverse event was cytokine release syndrome. Any-grade CRS was reported by 68% of autoimmune disease studies and 45% of infectious disease studies. However, severe CRS was less frequent, with grade ≥ 3 CRS reported by 8% of autoimmune disease studies and 12% of infectious disease studies.

Neurotoxicity was reported less frequently than CRS, reported by 5% of autoimmune disease studies and 8% of infectious disease studies. Cytopenias were relatively common in both categories, reported in 42% of autoimmune disease studies and 38% of infectious disease studies, likely reflecting the effects of lymphodepleting conditioning regimens and CAR T-associated hematologic toxicity.

Infectious complications were reported in 28% of autoimmune disease studies and 35% of infectious disease studies. This higher frequency observed in infectious disease studies may reflect baseline immune dysfunction, chronic viral infection, a history of antiviral treatment, or immune perturbation after CAR T cell infusion. Hypogammaglobulinemia and B cell aplasia were more frequent in autoimmune disease studies, consistent with the predominance of B-lineage-directed CAR T cell strategies. Hypogammaglobulinemia occurred in 52% of autoimmune disease studies compared with 30% of infectious disease studies, and B cell aplasia occurred in 65% of autoimmune disease studies compared with 28% of infectious disease studies.

**Figure 7:**
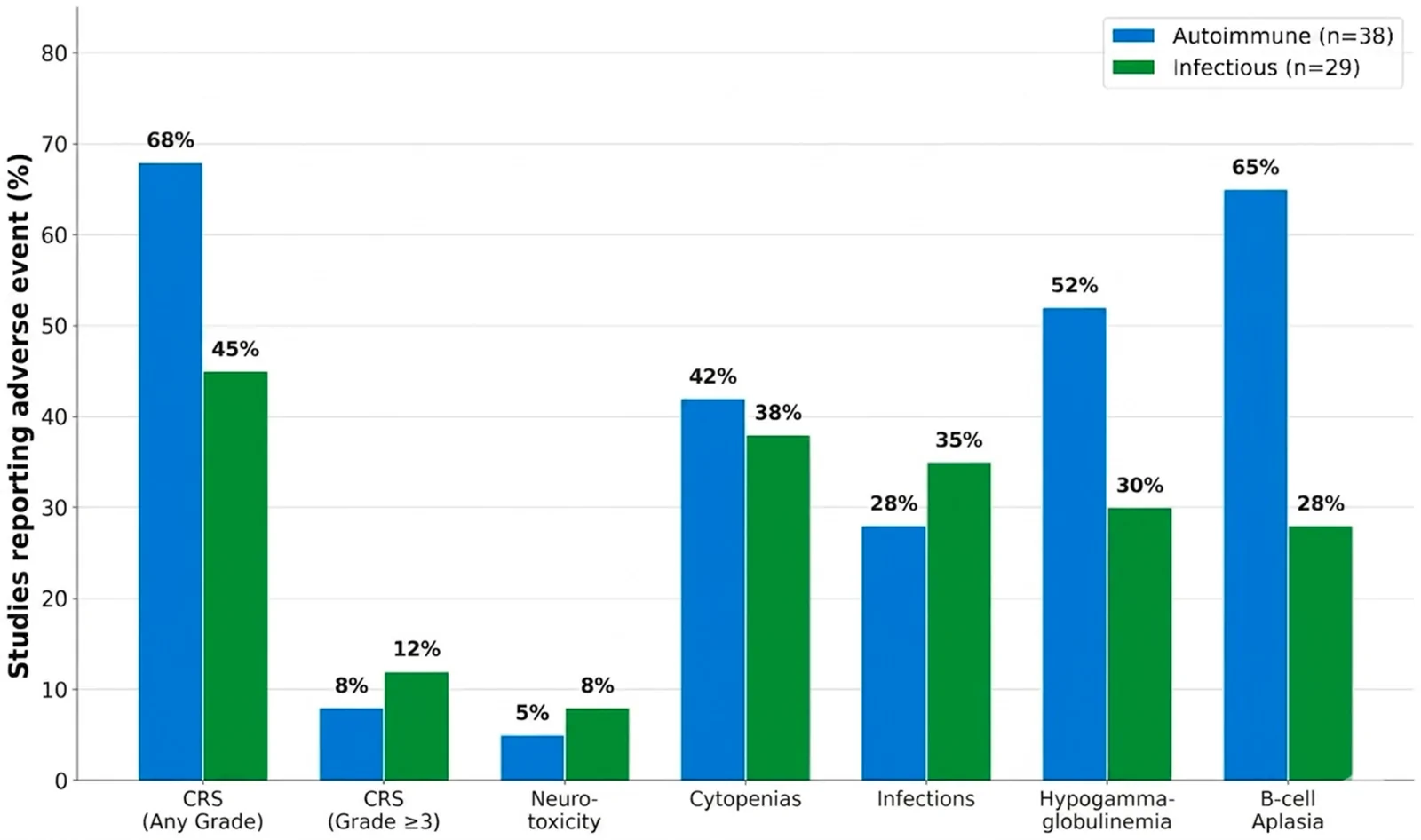
Descriptive frequencies of safety reports by disease domain. The figure summarizes the proportion of autoimmune and infectious disease studies reporting each adverse-event category. Values represent study-level reporting frequencies and should not be interpreted as pooled patient-level adverse-event incidence rates. Clinical studies were prioritized for patient-safety interpretation, whereas preclinical and translational studies were used only for mechanistic safety context. Because adverse-event definitions, grading, follow-up duration, and denominator reporting varied across studies, no formal pooled safety meta-analysis was performed.

These safety findings suggest that non-oncological CAR T cell therapy may be feasible, but careful monitoring remains essential. Major long-term safety concerns include prolonged immunosuppression, delayed B cell recovery, hypogammaglobulinemia, infection risk, and the uncertain durability of immune reconstitution. Safety outcomes were interpreted primarily from human clinical studies. Preclinical, in vitro, and platform-development studies were not used to infer patient-level toxicity rates. The safety values shown in Figure 7 represent study-level reporting frequencies rather than pooled patient-level adverse-event incidence rates. The source data supporting this figure are provided in Supplementary Table S5A. Descriptive source data summary for Figure 7 safety reporting frequencies by adverse event category and disease domain are shown in S5B.

## 4. Discussion

This systematic review indicates that CAR T cell therapy beyond oncology is emerging as a distinct immune-engineering strategy rather than a simple extension of cancer-directed cellular therapy. The reviewed evidence shows that non-oncological CAR T cell applications differ from oncology in therapeutic intent, target selection, acceptable toxicity thresholds, and expected durability of immune reprogramming [1,2,4,6,10,69,70]. Across the included studies, the most mature early clinical signals were observed in selected patients with severe refractory autoimmune diseases, particularly B cell-mediated systemic autoimmune disorders such as systemic lupus erythematosus (SLE), systemic sclerosis, and inflammatory myopathies [14,16,36,43]. In contrast, infectious disease applications remain less clinically mature and are supported mainly by early-phase, translational, and preclinical studies targeting HIV, HBV, HCV, CMV, and EBV-associated disease [17,18,19,20,21,22]. Therefore, the current evidence suggests a stronger and more clinically advanced therapeutic signal in autoimmune diseases than in infectious diseases, although both fields remain limited by small cohorts, heterogeneous endpoints, and short follow-up [25,28,33,39,68].

The synthesis emphasizes that the biological meaning of a response differs substantially across indications. In B cell-mediated autoimmune disease, response may reflect depletion of autoreactive B cell compartments and subsequent immune reconstitution. In HIV, response is constrained by antigen-silent reservoirs, viral escape, and the potential susceptibility of the engineered T cell product to infection. In HBV, target recognition must be balanced against the risk of immune-mediated liver injury. For CAR-Treg approaches, the central question is not cytotoxic potency but whether the engineered cells retain regulatory identity, traffic to the relevant tissue, and induce durable antigen-specific tolerance without inflammatory conversion.

A key interpretive consideration is that clinical and nonclinical evidence cannot be weighted equally. The most clinically relevant evidence in this review comes from human studies, particularly early clinical reports and small prospective cohorts from severe, refractory autoimmune diseases. These studies support preliminary clinical activity but remain limited by small sample sizes, uncontrolled designs, selected patient populations, and short follow-up periods. In contrast, animal, in vitro, and platform development studies provide important mechanistic and engineering insights but cannot establish clinical efficacy. Therefore, conclusions about efficacy in this review are restricted to human clinical data, while preclinical evidence is used to explain biological plausibility and guide future study design.

In autoimmune diseases, the main therapeutic concept is immune reset rather than continuous immunosuppression. In early reports, CD19-directed CAR T cell therapy has been associated in early reports with profound B cell depletion, reductions in autoantibodies, improvements in complement levels, steroid withdrawal, and drug-free remission in selected patients with severe, refractory SLE and related systemic autoimmune diseases [11,13,16,36,37,43]. Mechanistically, these findings support the hypothesis that CAR T cells may eliminate pathogenic B cell compartments more deeply than conventional B cell-depleting monoclonal antibodies, allowing immune reconstitution from naïve B cells with a less autoreactive profile [12,14]. Compared with standard immunosuppressive or biologic therapies, this suggests a more durable immunological effect. However, this interpretation remains preliminary and requires confirmation in larger prospective studies with longer follow-up, standardized immune-monitoring protocols, and clearly defined relapse criteria [25,28,32,39].

The strength of evidence varies substantially between disease areas. Autoimmune disease studies, particularly those involving CD19-directed CAR T cells in severe refractory SLE, provide the most persuasive early clinical signal [13,15,16]. However, many reports remain small, uncontrolled, and enriched for highly selected patients who were eligible for lymphodepletion and cellular therapy [25,28,39]. Consequently, clinical improvement cannot be attributed with certainty to CAR T cells alone. Lymphodepleting chemotherapy, intensified monitoring, supportive care, regression to the mean, and natural fluctuation in autoimmune disease activity may also contribute to the observed benefit. Controlled prospective trials comparing CAR T cell therapy with the best available biologic or targeted therapy will therefore be essential, although such trials may be ethically and logistically difficult in rapidly progressive or organ-threatening autoimmune disease [32,33].

Compared with autoimmune diseases, infectious disease applications present a more complex biological and translational challenge. HIV-directed CAR T cell therapy illustrates both the promise and limitations of pathogen-directed cellular therapy. CAR T cells can be engineered to recognize HIV envelope proteins and may be combined with protective strategies such as CCR5 disruption, multispecific antigen recognition, or broadly neutralizing antibody-derived CAR designs [18,19,20]. However, the clinically relevant HIV reservoir is often latent and antigen-silent, which limits CAR T cell recognition unless latency is reversed. Therefore, CAR T cell therapy alone is unlikely to eradicate HIV. A more realistic strategy may require combination approaches involving latency-reversing agents, broadly neutralizing antibodies, therapeutic vaccination, immune checkpoint modulation, and gene-editing technologies [6,7,19,27,40].

HBV-directed CAR T cell therapy presents a different challenge. Antigen recognition is biologically feasible because CAR T cells can be redirected against HBV envelope or surface antigens [21,22]. However, the target tissue is the liver, where excessive immune-mediated cytotoxicity may cause clinically significant hepatitis. Unlike malignant B cells, hepatocytes are not expendable. Therefore, disease-specific safety engineering is particularly important for HBV and other organ-tropic infections. Potential approaches include transient mRNA CAR expression, suicide switches, dose fractionation, affinity-tuned receptors, local delivery, or synthetic control systems that limit CAR activity and reduce the risk of uncontrolled tissue injury [8,21,22,40]. To place the findings in context, we compared antigenic targets and construct designs across the major disease areas. CD19- and BCMA-directed CAR-T cells were considered best suited to B cell-mediated autoimmune diseases because they can achieve deep depletion of pathogenic naïve, memory, and plasma cell compartments, thereby supporting an “immune reset” [13,14,15,16]. This rationale is supported by early clinical reports of anti-CD19 CAR-T therapy for severe, refractory systemic lupus erythematosus and other autoimmune diseases [14]. By contrast, HIV-directed applications require multi-specific, protected or reservoir-aware constructs, such as those based on broadly neutralizing antibodies, dual targeting of gp120/CD4, CCR5 disruption or other protective engineering, because single-target constructs are susceptible to viral escape and may render the engineered T cell itself vulnerable to infection [18,20]. Hepatitis B and C virus applications were emphasized as requiring transient, dose-controllable, or safety-switch constructs because the liver is not an expendable organ; persistent high-potency cytotoxic activity could cause clinically significant hepatitis or liver injury [21,22]. Finally, we distinguished cytotoxic CAR-T platforms from CAR-Treg platforms whose goal is antigen-specific immune regulation rather than cell killing, noting that the design of CAR-Tregs must prioritize stable FOXP3 expression, tissue trafficking, and resistance to inflammatory conversion rather than maximal cytotoxic or proliferative effector function, which is the design priority for conventional (cytotoxic) CAR T cells but would be counterproductive for a suppressive CAR-Treg product [36,37].

CAR-Tregs represent a parallel, conceptually distinct non-oncological CAR platform, as many immune-mediated diseases require tolerance induction rather than cytotoxic depletion. This approach is particularly attractive in transplantation, autoimmune disease, allergy, inflammatory bowel disease, and neuroinflammation because CAR-Tregs are designed to suppress immune responses in an antigen-directed manner [23,26]. Compared with cytotoxic CAR T cells, CAR-Tregs may offer a safer and more physiologically targeted strategy for diseases in which immune regulation, rather than immune elimination, is required. However, translation remains in its early stages. In transplantation, defined alloantigens may facilitate target selection, whereas in autoimmune diseases, identifying tissue-relevant and disease-specific antigens remains more difficult. A central challenge for CAR-Treg development is lineage stability, as loss of regulatory identity could render the product ineffective or pro-inflammatory. Future clinical development should therefore include molecular identity testing, epigenetic stability assessment, suppressive function assays, trafficking analysis, and long-term monitoring for inflammatory conversion [5,7,8,26,40].

The evidence was then organized by disease maturity and level of evidence. Among the autoimmune indications, severe refractory systemic lupus erythematosus (with or without lupus nephritis) currently has the strongest early clinical signal, with small cohorts and case series demonstrating rapid serological improvement, durable remission, and immune reconstitution [13]. Rheumatoid arthritis and multiple sclerosis show encouraging but less consistent results and currently lack long-term follow-up. Systemic sclerosis, inflammatory myopathies, and other rare autoimmune syndromes are represented by only a handful of cases [49,50,51,71]. In contrast, type 1 diabetes, inflammatory bowel disease, and myasthenia gravis remain at pre-clinical or translational stages, with primarily mechanistic or tolerogenic CAR-Treg approaches reported [37,39]. Infectious-disease applications, including HIV, HBV, HCV, CMV, EBV, and tuberculosis, are also at early-phase or pre-clinical stages [17,20]. CAR-Treg therapies likewise remain proof-of-concept, with no clinical trials yet demonstrating definitive efficacy [36,37].

Durability and relapse were then considered in light of B cell repopulation, maintenance-therapy withdrawal, and retreatment. The available data suggest that long-term benefit depends on durable B cell depletion followed by repopulation from a less autoreactive B cell repertoire. However, follow-up beyond 12 months is sparse, and relapse rates remain uncertain in most indications [13]. Reports of re-treatment or a second course of CAR-T therapy are rare and should be regarded as investigational [48]. Maintenance immunosuppression has been successfully discontinued in some cases of systemic lupus erythematosus, but it is premature to generalize this strategy beyond carefully selected responders [13,44,45,48]. In infectious-disease applications, discontinuing antiretroviral or antiviral therapy after CAR-T treatment is not yet supported and could lead to rebound viremia [17,20]. Persistent hypogammaglobulinemia, infection risk, and organ-specific toxicities (for example, hepatic flares in HBV) must be monitored as potential barriers to durable benefit [45].

A recurring theme across all indications is target selection. In oncology, on-target/off-tumor depletion of normal B cells is often accepted because B cell aplasia can be clinically managed. Outside oncology, this risk–benefit logic is less straightforward. Patients with autoimmune or infectious diseases may be younger, may have longer life expectancies, and may require decades of immune competence. The ideal non-oncological CAR target should therefore be disease-enriched, functionally relevant, safely depletable or regulatable, and linked to measurable biomarkers of response [13,14,15,16,22]. Where such targets are unavailable, more sophisticated engineering approaches may be required, including logic-gated CARs, synNotch systems, dual-antigen recognition, transient CAR expression, local delivery, regulated ON/OFF switches, and allogeneic or in vivo CAR platforms [5,7,8,40]. We also defined immune-system reset as a separate biological outcome distinct from clinical improvement. Evidence considered supportive of immune reset includes deep depletion of pathogenic B cell compartments, disappearance or reduction in disease-associated autoantibodies, normalization of complement, recovery of naïve B cell repertoires, discontinuation of maintenance immunosuppression, and sustained remission after immune reconstitution [13,41,45,48,72]. This phenomenon is best documented in CD19-directed CAR-T therapy for severe, refractory systemic lupus erythematosus, where anti-double-stranded DNA antibody titers decline, complement levels recover, and naïve B cell repopulation occurs [13,16,45,48]. Other autoimmune diseases show partial signals, but larger studies are needed [46,72]. Evidence for an immune reset is currently lacking in infectious-disease and CAR-Treg applications [17,36,37].

Manufacturing remains a major translational barrier. Autologous CAR T cell therapy is individualized, expensive, time-consuming, and technically demanding. Patients with autoimmune diseases often receive corticosteroids, biologics, or other immunosuppressive agents that may affect T cell fitness, expansion, and product consistency. Patients with chronic infectious diseases may have exhausted, dysfunctional, or infected T cell compartments [17,22,25,28,39]. Allogeneic off-the-shelf CAR T products, induced pluripotent stem cell-derived immune cells, automated closed-system manufacturing, and in vivo CAR programming may improve scalability and access. However, these approaches introduce additional safety concerns, including graft-versus-host disease, immune rejection, genome-editing risks, persistence control, and long-term surveillance requirements [5,6,7,8,9].

Regulatory and ethical considerations are especially important for non-malignant disease indications. In oncology, higher toxicity may be acceptable in the context of imminent mortality or lack of curative options. In non-malignant diseases, the benefit–to–risk threshold is more complex. Some autoimmune diseases are severe, organ-threatening, and life-shortening, whereas others are chronic but manageable with existing therapies. At present, CAR T cell therapy should be considered mainly for patients with severe, refractory, or organ-threatening disease until stronger long-term safety and comparative effectiveness data are available [25,28,32,33,68]. As the evidence matures, the field will need to determine whether CAR T cell therapy should remain a rescue therapy or be introduced earlier to prevent irreversible organ damage.

Finally, we outlined several unresolved mechanistic questions. These include how CAR-T persistence and memory formation relate to relapse or loss of response; how antigen density, tissue compartmentalization, and microenvironmental factors influence efficacy and toxicity; how CAR-Tregs maintain lineage stability and avoid inflammatory reprogramming in vivo; and how to balance transient versus durable CAR expression for indications such as HBV, where target-cell depletion must be reversible [6,8,36,37]. We emphasized the need for longer follow-up, standardized endpoint definitions, consistent reporting of relapse, retreatment, immune reset, and quality-of-life outcomes, and mechanistic studies that distinguish target-cell depletion from immune regulation [1,23]. Addressing these questions will be essential to optimizing CAR-T therapy beyond oncology [10,28].

The timing of CAR T cell therapy should therefore be interpreted cautiously. In the available clinical evidence, CAR T cell therapy was generally used after multiple standard therapies had failed or in severe, organ-threatening disease. This late positioning reflects the current evidence base and ethical threshold, not proof that CAR T cells are biologically effective only at the end stage. Future trials should test whether earlier use in carefully selected high-risk patients can prevent irreversible damage while maintaining an acceptable safety profile.

Outcome standardization is urgently needed. Autoimmune studies should consistently report disease-specific activity scores, organ involvement, steroid dose, immunosuppressive withdrawal, organ damage indices, patient-reported outcomes, autoantibody titers, complement levels, B cell subsets, immunoglobulin levels, infections, relapse definitions, and retreatment [13,36,37]. Infectious disease studies should report viral load, reservoir assays, tissue sampling where feasible, antiviral therapy status, analytical criteria for treatment interruption, immune escape, and markers of immune exhaustion [18,20,22]. Across all disease settings, CAR expansion, persistence, phenotype, vector copy number, manufacturing characteristics, lymphodepletion regimen, dose, adverse events, and long-term immune reconstitution should be reported transparently to enable meaningful comparisons across studies [30,32,68,70].

The health-economic implications also require careful consideration. Current CAR T cell therapy is costly, and broad application to prevalent autoimmune or infectious diseases would not be sustainable without manufacturing innovation, patient-selection criteria, and long-term value assessment [5,6,7,8,33,40]. However, cost evaluation should account for the cumulative burden of severe refractory disease, including repeated hospitalizations, chronic biologic therapy, disability, organ failure, dialysis, transplantation, opportunistic infections, and productivity loss. A one-time high-cost therapy may become cost-effective if it induces durable remission, prevents irreversible organ damage, and reduces long-term healthcare utilization. Robust cost-effectiveness analyses with long-term real-world follow-up are therefore needed before CAR T cell therapy can be widely implemented in non-malignant diseases [25,28,70].

### 4.1. Limitations of the Current Evidence and of This Review

The current evidence base has several important limitations. First, most clinical studies remain small, early-phase, uncontrolled, and enriched for highly selected patients. This limits causal inference and reduces generalizability to broader populations of autoimmune and infectious diseases. Second, follow-up remains short relative to the lifelong nature of many autoimmune and chronic infectious diseases. Late relapse, delayed immune deficiency, secondary malignancy risk, fertility effects, vaccination response, long-term infection risk, and quality-of-life outcomes remain insufficiently characterized.

Third, outcome heterogeneity was substantial. Studies used different disease activity scores, definitions of remission, biomarker panels, viral reservoir assays, CAR persistence measures, and toxicity reporting systems. This heterogeneity prevented formal meta-analysis and limited the ability to compare outcomes across diseases, CAR constructs, and study designs. Therefore, the findings of this review should be interpreted as a structured descriptive synthesis rather than a quantitative estimate of comparative efficacy.

Fourth, adverse-event reporting was inconsistent across studies. Some studies reported patient-level toxicity data, whereas others reported only selected events or qualitative safety summaries. As a result, adverse-event frequencies in this review should not be interpreted as pooled patient-level incidence rates unless explicitly stated. Fifth, manufacturing details were often incompletely reported, including T cell phenotype, CD4:CD8 composition, transduction efficiency, vector copy number, expansion time, viability, exhaustion markers, release criteria, and product comparability. These variables may strongly influence efficacy, persistence, and toxicity.

Sixth, infectious disease studies frequently relied on surrogate endpoints such as viral load, reservoir assays, antigen expression, or in vitro cytotoxicity. Although these measures are biologically relevant, they do not necessarily establish functional cure, durable pathogen clearance, or long-term clinical benefit. Seventh, preclinical CAR-Treg studies may overestimate translational success because animal models do not fully reproduce human disease heterogeneity, chronicity, immune history, and treatment exposure. Eighth, publication bias is likely because dramatic responses and successful early applications are more likely to be reported than non-responses, relapses, manufacturing failures, or late toxicity.

This review also has limitations. Although a systematic search strategy was used, the field is rapidly evolving, and new trial results, conference abstracts, registry updates, and regulatory developments continue to emerge. Some relevant data may remain unpublished or available only in interim form. In addition, the inclusion of clinical, preclinical, and translational studies broadened the review but also introduced substantial heterogeneity in the level of evidence. These study types were therefore interpreted separately, with clinical evidence prioritized for therapeutic conclusions and preclinical evidence used mainly to support mechanistic rationale and future translational directions.

### 4.2. Future Directions

Future development of CAR T cell therapy beyond oncology should be guided by several priorities. First, larger multicenter prospective trials are needed in the most promising autoimmune indications, particularly systemic lupus erythematosus, systemic sclerosis, and inflammatory myopathies. These trials should include standardized eligibility criteria, disease-specific and organ-specific end-points, steroid-sparing outcomes, patient-reported outcomes, immune reconstitution profiling, and long-term safety monitoring.

Second, combination strategies should be rationally designed according to disease biology. In autoimmune diseases, bridging therapy and lymphodepletion intensity should be optimized to reduce the inflammatory burden while minimizing cytopenias, infections, and prolonged immunosuppression. In HIV, CAR T cells will likely need to be combined with latency reversal, broadly neutralizing antibodies, therapeutic vaccines, immune checkpoint modulation, or gene editing to address antigen-silent reservoirs. In HBV, antiviral therapy should be integrated to suppress replication and reduce liver injury during CAR-mediated immune targeting.

Third, safer CAR designs are required for non-malignant indications. These may include suicide switches, transient mRNA CAR expression, regulated ON/OFF systems, logic-gated CARs, affinity-tuned receptors, dual-antigen recognition, tissue-restricted activation, and local delivery strategies. These safety systems are particularly important where the target tissue is not expendable or where long-term immune competence is essential.

Fourth, CAR-Treg development should focus on lineage stability, antigen specificity, tissue trafficking, persistence, suppressive potency, and validated release criteria. Future studies should incorporate molecular and epigenetic assays that confirm maintenance of regulatory identity and reduce the risk of inflammatory conversion after infusion.

Fifth, manufacturing innovation is essential. Autologous products may remain important for early proof of concept and individualized therapy, but broader clinical implementation will require faster, less expensive, and more consistent platforms. Allogeneic CAR T cells, in vivo engineering, automated closed-system manufacturing, and induced pluripotent stem cell-derived immune-cell platforms may improve scalability if their safety, persistence, and controllability are demonstrated.

Sixth, international long-term registries should be established for all non-oncology CAR T cell recipients. Minimum datasets should include disease activity, relapse, retreatment, infections, immunoglobulin levels, vaccination response, fertility outcomes, secondary malignancies, quality of life, survival, CAR persistence, and late immune complications. Such registries will be indispensable for defining the true benefit–risk profile of CAR T cell therapy in non-malignant diseases. Future clinical trials should prioritize disease-specific endpoints, standardized definitions of remission and relapse, longer follow-up, and systematic immune monitoring. Future studies should also be prospectively registered and should use harmonized minimum reporting datasets to enable cross-study comparison of efficacy, toxicity, immune reconstitution, CAR persistence, and long-term relapse. Such harmonization will be essential for determining which non-oncological indications are most suitable for CAR T cell therapy and for distinguishing true disease modification from transient clinical improvement.

## 5. Conclusions

CAR T cell therapy is entering a new phase in which its potential value extends beyond cytotoxicity against cancer. In autoimmune diseases, especially severe refractory systemic lupus erythematosus and related systemic autoimmune disorders, CD19-directed CAR T cell therapy has generated encouraging early evidence of deep B cell depletion, serological improvement, steroid withdrawal, and drug-free remission in selected patients. These findings support the concept of immune reset, but they remain preliminary and require validation in larger controlled studies with longer follow-up. Clinical efficacy conclusions were based primarily on human studies, whereas preclinical and translational evidence was used to support mechanistic interpretation and identify future development priorities.

In infectious diseases, CAR T cell therapy remains less clinically mature but offers a rational strategy to target persistent viral reservoirs and restore pathogen-directed cellular immunity. Current evidence is strongest at the mechanistic and early translational levels, and durable pathogen eradication has not yet been established. CAR-Tregs further broaden the field by enabling antigen-specific immune tolerance rather than immune destruction, although clinical validation remains limited.

Overall, broad clinical adoption of CAR T cell therapy for non-malignant diseases remains premature. This review retains its classification as a systematic review because it uses a predefined PICOS framework, systematic searches of databases and registries, duplicate removal, two-reviewer screening, eligibility criteria, structured data extraction, risk-of-bias assessment, and PRISMA reporting. The synthesis is descriptive rather than meta-analytic because the retrieved evidence is heterogeneous across disease indications, CAR constructs, endpoint definitions, and study designs. This descriptive synthesis should not be confused with a bibliometric analysis, as the review does not primarily quantify publication output or citation patterns; instead, it systematically extracts and interprets therapeutic, immunological, virological, safety, and translational outcomes.

## Figures and Tables

**Table 1:** PICOS framework is used to define the systematic review question and eligibility criteria.

PICOS Element	Definition Used in This Review
Population	Patients, animal models, or translational models of autoimmune or infectious diseases.
Intervention	CAR T cells, CAR-Tregs, autologous or allogeneic CAR platforms, and closely related engineered CAR T cell approaches.
Comparator	Standard therapy, untreated controls, baseline status, disease controls, non-CAR controls, or no comparator, depending on study design.
Outcomes	Clinical remission, disease activity improvement, biomarker improvement, immune reconstitution, viral load or reservoir reduction, CAR T cell expansion/persistence, relapse, retreatment, and adverse events.
Study design	Clinical trials, prospective or retrospective cohorts, case series, preclinical animal studies, and translational investigations.

**Table 2:** Data-extraction framework and source-data fields used for the study-level evidence matrix and descriptive figures.

Variable Group	Fields Recorded in the Study-Level Evidence Matrix
Study identification	First author, year, country, citation number, database source, clinical trial or registry identifier where available
Study design and population	Clinical, in vivo animal, in vitro/translational, or platform-development status; sample size; disease indication; inclusion/exclusion features; prior therapy; follow-up duration
CAR construct	CAR target antigen, binding-domain source, CAR generation, costimulatory domain, vector or RNA platform, autologous/allogeneic cell source, lymphodepletion, dose
Effectiveness outcomes	Disease-specific response/remission definition, biomarker response, viral or reservoir response, relapse, retreatment, maintenance-therapy withdrawal
Safety outcomes	CRS, ICANS/neurotoxicity, cytopenias, infection, hypogammaglobulinemia, organ toxicity, treatment-related mortality
Quality appraisal	Risk-of-bias or quality-assessment tool applied according to design; main limitations affecting interpretation
**Source-Data Field-1**	**Definition**
Disease-target category	Disease indication and CAR target used to group studies
Contributing studies	Citation numbers and number of studies contributing extractable response data
Evaluable denominator	Number of treated participants or experimental units with response data
Response numerator	Number meeting the response/remission definition used by the original study
Reported range	Study-level range when more than one study contributed; not a confidence interval
Interpretation	Descriptive response signal; not a pooled comparative efficacy estimate
**Source-Data Field-2**	**Definition**
Adverse-event category	CRS, ICANS/neurotoxicity, cytopenias, infection, hypogammaglobulinemia, organ toxicity, or another reported event
Evidence type	Clinical human study, preclinical animal study, or in vitro/translational report
Contributing studies	Number and citation numbers of studies reporting the event category
Evaluable participants	Number of treated participants contributing clinical safety data, where available
Reporting frequency	Study-level reporting frequency; not pooled patient-level incidence
Interpretation	Used to identify safety themes and reporting gaps, not to compare incidence across diseases

**Table 4:** A clinical-study-focused extraction and synthesis framework is used to distinguish clinical, in vivo, in vitro/translational, and CAR-Treg evidence. Abbreviation: CRS, cytokine release syndrome.

Evidence Domain	Predominant Study Type Retained for Detailed Synthesis	Representative CAR Approach	Key Extracted Outcomes	Interpretation in the Systematic Review
SLE/lupus nephritis	Early clinical reports and small prospective cohorts	CD19 CAR T cells after lymphodepletion	Disease activity/remission, anti-dsDNA, complement recovery, steroid/immunosuppressant withdrawal, relapse, CRS, cytopenias, infection, hypogammaglobulinemia	Strongest early clinical signal; still preliminary because cohorts are small and mostly uncontrolled.
Systemic sclerosis/inflammatory myopathies	Clinical case series and translational reports	Mainly CD19 CAR T cells	Organ-specific response, autoantibody change, functional improvement, treatment-free interval, CRS, cytopenias, infection	Promising but preliminary evidence requiring standardized endpoints and longer follow-up.
Other autoimmune diseases including RA, MS, T1D, IBD and MG	Mostly preclinical, translational, or early clinical evidence	CD19, BCMA, CAAR, CAR-Treg, and disease-specific experimental constructs	Disease activity, antigen-specific immune modulation, tissue inflammation, relapse/retreatment, lineage stability for CAR-Tregs	Used mainly for mechanistic and translational synthesis unless clinical data were available.
HIV infection	Early phase clinical, translational, and preclinical studies	HIV envelope/CD4-derived CARs, dual-targeting or protected CAR T cells	Viral load, reservoir measures, CAR persistence, analytical treatment interruption where reported, immune escape	Supports feasibility and persistence signals, but not durable eradication.
HBV/HCV infection	Preclinical, translational, and limited clinical evidence	HBV surface/envelope-targeted CAR T cells and antiviral CAR designs	Viral antigen reduction, infected-cell killing, viral load or surrogate reservoir markers, liver enzyme elevation/hepatitis risk	Biologically rational but safety-limited because the target tissue is not expendable.
CAR-Treg platforms	Preclinical and early translational evidence	Antigen-specific CAR-Tregs	Suppressive function, tissue trafficking, phenotype stability, tolerance markers, risk of inflammatory conversion	Analyzed separately because the therapeutic intent is tolerance rather than cytotoxicity.

**Table 7:** Expanded evidence-synthesis framework for non-oncological CAR T cell therapy: persistence, retreatment, treatment withdrawal, immune reset, intervention timing, and off-the-shelf platforms.

Question Added to Synthesis	Evidence Extracted	Main Finding	Implication
Persistence and survival of CAR T cells	Expansion peak, detectable CAR copies/cells, duration of B cell aplasia or target-cell depletion, follow-up duration	Persistence varied by construct and disease context; 4-1BB-containing constructs were generally interpreted as favoring longer persistence than CD28-dominant rapid-effector designs.	Persistence must be reported separately from clinical response; durable detectability is not equivalent to durable disease control.
Second course or retreatment	Relapse, B cell or pathogen-target recovery, repeat infusion, and retreatment after response loss	Evidence for routine second-course CAR T therapy outside oncology remains sparse; retreatment should be considered investigational and individualized.	Future studies should predefine relapse and retreatment criteria.
Stopping maintenance therapy	Steroid withdrawal, discontinuation of immunosuppressants, antiviral continuation, and drug-free remission	Autoimmune reports, especially SLE, provide the clearest signal that maintenance immunosuppression may be stopped in selected responders. Infectious disease studies do not yet support stopping antiviral therapy as a cure strategy.	Disease-specific post-CAR therapy rules are needed before broad implementation.
Immune reset	Autoantibody reduction, complement recovery, naive B cell reconstitution, memory B cell depletion, immunoglobulin recovery, and relapse status	Evidence for immune reset is strongest in B cell-mediated autoimmune disease, but remains based on small and selected cohorts.	Immune reset should be treated as a measurable biological outcome, not only as a conceptual claim.
Timing of intervention	Prior therapies, refractory status, organ-threatening disease, and baseline risk-benefit context	Most evidence comes from severe refractory patients after failure of standard modalities; this reflects the available evidence, not necessarily the biologically optimal timing.	Earlier use should be tested only in controlled trials with strict eligibility and safety monitoring.
Off-the-shelf CAR T	Allogeneic constructs, including gene-edited ‘universal’ platforms; gene editing, graft-versus-host risk, rejection, persistence control	Off-the-shelf products may improve scalability, but direct non-oncology evidence remains limited.	They should be discussed as translational opportunities rather than established clinical options.

## Data Availability

No new data were created or analyzed in this study. Data sharing is not applicable.

## Supplementary Materials

The following supporting information can be downloaded at: https://www.mdpi.com/article/10.3390/pathophysiology33030070/s1, Supplementary Table S1: PRISMA 2020 checklist; Supplementary Table S2: database-specific search strategies; Supplementary Table S3: aggregate source-data summary for Figure 5, disease-distribution counts; Supplementary Table S4A: aggregate source-data summary for Figure 6, CAR T cell construct-generation distribution; Supplementary Table S4B: representative construct designs and disease-specific rationale in non-oncological CAR T cell therapy; Supplementary Table S5A: descriptive source-data summary for Figure 7, response endpoints by disease indication and CAR T cell target; Supplementary Table S5B: descriptive source-data summary for Figure 6, safety reporting frequencies by adverse-event category and disease domain.

## Abbreviations

The following abbreviations are used in this manuscript:

AIDS	Acquired immunodeficiency syndrome
BCMA	B cell maturation antigen
CAR	Chimeric antigen receptor
CAR-NK	Chimeric antigen receptor natural killer cell
CAR-Treg	Chimeric antigen receptor regulatory T cell
CD	Cluster of differentiation
CMV	Cytomegalovirus
CRS	Cytokine release syndrome
DNA	Deoxyribonucleic acid
EBV	Epstein–Barr virus
GRADE	Grading of Recommendations Assessment, Development and Evaluation
GVHD	Graft-versus-host disease
HBV	Hepatitis B virus
HCV	Hepatitis C virus
HIV	Human immunodeficiency virus
ICANS	Immune effector cell-associated neurotoxicity syndrome
IBD	Inflammatory bowel disease
MHC	Major histocompatibility complex
NK	Natural killer
NOS	Newcastle–Ottawa Scale
PBMC	Peripheral blood mononuclear cell
PICOS	Population, Intervention, Comparator, Outcomes, and Study design
PRISMA	Preferred Reporting Items for Systematic Reviews and Meta-Analyses
RA	Rheumatoid arthritis
ROBINS-I	Risk Of Bias In Non-randomized Studies of Interventions
SLE	Systemic lupus erythematosus
SYRCLE	Systematic Review Centre for Laboratory Animal Experimentation
TCR	T cell receptor
TIL	Tumor-infiltrating lymphocyte
WHO ICTRP	World Health Organization International Clinical Trials Registry Platform
