# Supplementary material for: Beyond Oncology: Exploring the Expanding Role of CAR T Cell Therapy in Autoimmune and Infectious Diseases—A Systematic Review
Source: Pathophysiology. 2026 Sep 19;33(3):70. doi: 10.3390/pathophysiology33030070 (PMC13610205; doi:10.3390/pathophysiology33030070)
Supplement: Supplementary file 1 [file pathophysiology-33-00070-s001.zip › pathophysiology-4500763-supplementary.pdf]

Supplementary Materials

Beyond Oncology: Exploring the Expanding Role of CAR T Cell Therapy in Autoimmune and Infectious Diseases—A Systematic Review

Zaher, K.A.; Alrahimi, J. *Pathophysiology*, 2026

Purpose of these supplementary materials. Tables S1-S5 provide reporting, search-strategy, aggregate source-data, construct-design, response-endpoint, and safety-reporting summaries supporting the systematic review. Tables S3-S5 are descriptive source-data summaries and should not be interpreted as formal meta-analysis tables. No pooled effect estimates, pooled confidence intervals, odds ratios, risk ratios, or heterogeneity statistics are presented.

Supplementary Table S1. PRISMA 2020 checklist summary.

Note: This table summarizes PRISMA 2020 reporting locations. It provides a practical checklist for the submitted review. The wording of the full PRISMA items should be interpreted in accordance with the official PRISMA 2020 statement.

Section and Topic	Item #	Checklist item	Location where item is reported
**TITLE**	**TITLE**	**TITLE**	
Title	1	Identify the report as a systematic review.	Title page, p. 1 ('Beyond Oncology: Exploring the Expanding Role of CAR T Cell Therapy in Autoimmune and Infectious Diseases—A Systematic Review').
**ABSTRACT**	**ABSTRACT**	**ABSTRACT**	
Abstract	2	See the PRISMA 2020 for Abstracts checklist.	Abstract, p. 1. Reports background/rationale, methods (PRISMA 2020 guidance; databases searched – PubMed/MEDLINE, Scopus, Web of Science, Cochrane Library, ClinicalTrials.gov, major trial registries; dates 1 Jan 2010–31 Mar 2026; eligibility), key results, and conclusions, consistent with the PRISMA 2020 for Abstracts checklist.
**INTRODUCTION**	**INTRODUCTION**	**INTRODUCTION**	
Rationale	3	Describe the rationale for the review in the context of existing knowledge.	Introduction, p. 1–3 (rationale for extending CAR T-cell therapy beyond oncology into autoimmune and infectious disease, including the limitations of standard immunosuppressive therapy and antiviral therapy in achieving durable remission/cure).

Section and Topic	Item #	Checklist item	Location where item is reported
Objectives	4	Provide an explicit statement of the objective(s) or question(s) the review addresses.	Introduction / Methods Section 2.1 'Study Design and Reporting Framework', p. 3: "The review question was formulated to evaluate whether CAR T cells, CAR-Tregs, or related engineered CAR T cell platforms have demonstrated therapeutic, immunological, virological, or safety-related benefits in non-malignant disease settings."
**METHODS**	**METHODS**	**METHODS**	
Eligibility criteria	5	Specify the inclusion and exclusion criteria for the review and how studies were grouped for the syntheses.	Methods, Section 2.3 'Eligibility Criteria', p. 5; PICOS framework, Table 1, p. 4.
Information sources	6	Specify all databases, registers, websites, organisations, reference lists and other sources searched or consulted to identify studies. Specify the date when each source was last searched or consulted.	Methods, Section 2.2 'Data Sources and Search Strategy', p. 4–5 (PubMed/MEDLINE, Scopus, Web of Science, Cochrane Library, ClinicalTrials.gov, WHO ICTRP; searched 1 Jan 2010–31 Mar 2026); Supplementary Table S2.
Search strategy	7	Present the full search strategies for all databases, registers and websites, including any filters and limits used.	Supplementary Table S2 (full database-specific search strategies); summarized in Methods Section 2.2, p. 4–5.
Selection process	8	Specify the methods used to decide whether a study met the inclusion criteria of the review, including how many reviewers screened each record and each report retrieved, whether they worked independently, and if applicable, details of automation tools used in the process.	Methods, Sections 2.3–2.4, p. 5–6 (Rayyan-based duplicate removal, title/abstract and full-text screening; disagreements resolved by discussion and consensus); Figure 3, p. 11–12. Note: the manuscript does not explicitly state the number of reviewers who screened each record or whether screening was independent — recommend confirming this (e.g., K.Z. and J.A., per Author Contributions, p. 31).
Data collection process	9	Specify the methods used to collect data from reports, including how many reviewers collected data from each report, whether they worked independently, any processes for obtaining or confirming data from study investigators, and if applicable, details of automation tools used in the process.	Methods, Section 2.5 'Data Extraction', p. 6; Table 2. Note: as with item 8, the manuscript does not explicitly state the number of reviewers who extracted data or whether independently — recommend adding this detail.

Section and Topic	Item #	Checklist item	Location where item is reported
Data items	10a	List and define all outcomes for which data were sought. Specify whether all results that were compatible with each outcome domain in each study were sought (e.g. for all measures, time points, analyses), and if not, the methods used to decide which results to collect.	Methods, Sections 2.5 (p. 6) and 2.7 (p. 7–8); Table 2, p. 6 (effectiveness outcomes – clinical response/remission, biomarker, viral/reservoir response, relapse, retreatment; safety outcomes – CRS, ICANS/neurotoxicity, cytopenias, infection, hypogammaglobulinemia, organ toxicity, treatment-related mortality). Outcomes were extracted per each study's own reported endpoint rather than harmonized to one universal endpoint (p. 8).
Data items	10b	List and define all other variables for which data were sought (e.g. participant and intervention characteristics, funding sources). Describe any assumptions made about any missing or unclear information.	Methods, Section 2.5, p. 6; Table 2 (study identification, design/population, CAR construct variables, prior therapy, follow-up duration, etc.). No explicit statement on handling of missing or unclear information.
Study risk of bias assessment	11	Specify the methods used to assess risk of bias in the included studies, including details of the tool(s) used, how many reviewers assessed each study and whether they worked independently, and if applicable, details of automation tools used in the process.	Methods, Section 2.6 'Risk of Bias and Quality Assessment', p. 7 (Cochrane RoB 2 for randomized trials [ref. 29]; Newcastle–Ottawa Scale for non-randomized/observational studies [ref. 30]; SYRCLE tool for preclinical animal studies). Note: number of reviewers / independence not explicitly stated.
Effect measures	12	Specify for each outcome the effect measure(s) (e.g. risk ratio, mean difference) used in the synthesis or presentation of results.	Methods, Section 2.7 'Data Synthesis and Statistical Analysis', p. 7: a formal meta-analysis was not conducted because of heterogeneity across disease indications, CAR constructs, designs, endpoints, and follow-up; findings were synthesized descriptively rather than as a pooled effect measure.
Synthesis methods	13a	Describe the processes used to decide which studies were eligible for each synthesis (e.g. tabulating the study intervention characteristics and comparing against the planned groups for each synthesis (item #5)).	Methods, Section 2.7, p. 7 (grouping by disease category/pathogen and by clinical vs. preclinical/translational evidence before synthesis); Table 4, p. 13.
Synthesis methods	13b	Describe any methods required to prepare the data for presentation or synthesis, such as handling of	Methods, Section 2.7, p. 7–8 (no pooled summary statistics; responses categorized descriptively – clinical, biomarker, virological, reservoir, or mechanistic – per

Section and Topic	Item #	Checklist item	Location where item is reported
		missing summary statistics, or data conversions.	each study's own definition rather than converted to a common metric).
Synthesis methods	13c	Describe any methods used to tabulate or visually display results of individual studies and syntheses.	Results, Sections 3.3–3.6 and Supplementary Tables S3–S5, using descriptive tables (Tables 3–7) and figures (Figures 1, 4, 5, 6) to display study-level and grouped results.
Synthesis methods	13d	Describe any methods used to synthesize results and provide a rationale for the choice(s). If meta-analysis was performed, describe the model(s), method(s) to identify the presence and extent of statistical heterogeneity, and software package(s) used.	Methods, Section 2.7, p. 7: no statistical model was used; rationale given is heterogeneity of indications, constructs, designs, and endpoints. No software package applicable, as no pooled statistical synthesis was performed.
Synthesis methods	13e	Describe any methods used to explore possible causes of heterogeneity among study results (e.g. subgroup analysis, meta-regression).	Not applicable – no meta-analysis or heterogeneity statistics (e.g., subgroup analysis, meta-regression) were performed; studies are instead grouped descriptively by disease category/pathogen and construct generation (Section 2.7, p. 7; Supplementary Table S4A).
Synthesis methods	13f	Describe any sensitivity analyses conducted to assess robustness of the synthesized results.	Not applicable – no sensitivity analyses were conducted, consistent with the descriptive (non-meta-analytic) synthesis approach (Section 2.7, p. 7).
Reporting bias assessment	14	Describe any methods used to assess risk of bias due to missing results in a synthesis (arising from reporting biases).	Not described as a distinct Methods step. Publication/reporting bias is instead acknowledged qualitatively as a limitation in the Discussion (p. 29: "publication bias is likely because dramatic responses and successful early applications are more likely to be reported…"). Recommend adding a brief statement in Section 2.7 noting that no formal reporting-bias assessment (e.g., funnel plot) was planned, given the descriptive design.
Certainty assessment	15	Describe any methods used to assess certainty (or confidence) in the body of evidence for an outcome.	Methods, Section 2.6, p. 7: formal GRADE certainty grading was not applied across all outcomes; certainty of evidence was instead described qualitatively (strong / moderate / limited / preliminary) based on study design, sample size, consistency, and directness of evidence.
**RESULTS**	**RESULTS**	**RESULTS**	

Section and Topic	Item #	Checklist item	Location where item is reported
Study selection	16a	Describe the results of the search and selection process, from the number of records identified in the search to the number of studies included in the review, ideally using a flow diagram.	Results, Section 3.2 'Study Selection', p. 11 (2,847 database + 213 other-source records identified; 2,218 screened after de-duplication; 1,876 excluded; 342 full-text articles assessed; 264 excluded; 78 studies included); PRISMA 2020 flow diagram, Figure 3, p. 11–12.
Study selection	16b	Cite studies that might appear to meet the inclusion criteria, but which were excluded, and explain why they were excluded.	Results, Section 3.2, p. 11 (264 full-text exclusions grouped by category: reviews/commentaries without original data; in vitro-only without translational relevance; insufficient extractable data; animal-only studies; duplicate cohort reports; case reports with fewer than three patients). Note: excluded studies are described by category rather than cited individually by reference number — recommend confirming whether a per-study exclusion list is available, since item 16b calls for the specific excluded studies to be cited.
Study characteristics	17	Cite each included study and present its characteristics.	Results, Section 3.3 'General Characteristics of the Included Studies', p. 12–13; Table 4, p. 13; Supplementary Table S3.
Risk of bias in studies	18	Present assessments of risk of bias for each included study.	Not presented as a dedicated Results subsection or per-study table; risk-of-bias methodology is described in Section 2.6 (p. 7), and overall quality/limitations are discussed narratively in the Discussion (p. 29–30). Recommend adding an explicit Results statement or table of per-study risk-of-bias ratings, since item 18 calls for these to be reported as a Results item.
Results of individual studies	19	For all outcomes, present, for each study: (a) summary statistics for each group (where appropriate) and (b) an effect estimate and its precision (e.g. confidence/credible interval), ideally using structured tables or plots.	Results, Sections 3.3–3.6, p. 12–23; study-level data summarized in Tables 4–7 (p. 13, 18, 21–22) and Supplementary Tables S3–S5.
Results of syntheses	20a	For each synthesis, briefly summarise the characteristics and risk of bias among contributing studies.	Results, Sections 3.3–3.6, p. 12–23 (study and risk-of-bias characteristics are discussed narratively alongside the descriptive synthesis for each disease

Section and Topic	Item #	Checklist item	Location where item is reported
			group, rather than in a separate per-synthesis summary table).
Results of syntheses	20b	Present results of all statistical syntheses conducted. If meta-analysis was done, present for each the summary estimate and its precision (e.g. confidence/credible interval) and measures of statistical heterogeneity. If comparing groups, describe the direction of the effect.	Results, Sections 3.5–3.6, p. 15–23; Tables 5–7 and Figures 5–6 (descriptive response estimates and ranges per disease/pathogen; explicitly labelled as descriptive, not pooled effect estimates – e.g., Table 6 footnote, p. 21: "Descriptive synthesis only; not a pooled effect estimate").
Results of syntheses	20c	Present results of all investigations of possible causes of heterogeneity among study results.	Not applicable – no formal investigation of heterogeneity (e.g., subgroup analysis, meta-regression) was performed, consistent with the descriptive synthesis approach (Section 2.7, p. 7).
Results of syntheses	20d	Present results of all sensitivity analyses conducted to assess the robustness of the synthesized results.	Not applicable – no sensitivity analyses were performed, consistent with the descriptive (non-meta-analytic) synthesis approach.
Reporting biases	21	Present assessments of risk of bias due to missing results (arising from reporting biases) for each synthesis assessed.	Discussion, p. 29 ("Eighth, publication bias is likely because dramatic responses and successful early applications are more likely to be reported than non-responses, relapses…"). Presented narratively as a limitation rather than as a per-synthesis assessment.
Certainty of evidence	22	Present assessments of certainty (or confidence) in the body of evidence for each outcome assessed.	Discussion, p. 24–30, incorporating the qualitative certainty descriptors (strong/moderate/limited/preliminary) introduced in Methods Section 2.6, p. 7; limitations affecting certainty are discussed on p. 29–30.
**DISCUSSION**	**DISCUSSION**	**DISCUSSION**	
Discussion	23a	Provide a general interpretation of the results in the context of other evidence.	Discussion, Section 4, p. 24–29 (interpretation of findings for autoimmune disease, infectious disease, and CAR-Treg applications in the context of existing oncology and non-oncology evidence).
Discussion	23b	Discuss any limitations of the evidence included in the review.	Discussion, p. 29–30 ("This review also has limitations…" – heterogeneity of study types, evolving/unpublished evidence, publication bias, and reliance on preclinical/translational evidence for mechanistic interpretation).

Section and Topic	Item #	Checklist item	Location where item is reported
Discussion	23c	Discuss any limitations of the review processes used.	Discussion, p. 30 (limitations of the review process itself – e.g., broad inclusion of clinical, preclinical, and translational studies introducing heterogeneity in level of evidence; no formal registration or protocol).
Discussion	23d	Discuss implications of the results for practice, policy, and future research.	Discussion, 'Future Directions' subsection, p. 30–31 (priorities for larger multicenter trials, combination strategies, safer CAR designs, CAR-Treg development, manufacturing innovation, and international long-term registries).
**OTHER INFORMATION**	**OTHER INFORMATION**	**OTHER INFORMATION**	
Registration and protocol	24a	Provide registration information for the review, including register name and registration number, or state that the review was not registered.	Methods, Section 2.1, p. 4: "This review was not prospectively registered…"
Registration and protocol	24b	Indicate where the review protocol can be accessed, or state that a protocol was not prepared.	Methods, Section 2.1, p. 4: "…and no separate review protocol is available."
Registration and protocol	24c	Describe and explain any amendments to information provided at registration or in the protocol.	Methods, Section 2.1, p. 4: "…therefore, all departures from a formal protocol were documented in the Methods section, including predefined eligibility criteria, extraction variables, and synthesis methods."
Support	25	Describe sources of financial or non-financial support for the review, and the role of the funders or sponsors in the review.	Funding, p. 31 ("This project is funded by the Deanship of Scientific Research (DSR) at King Abdulaziz University, Jeddah, Saudi Arabia, under grant no. IPP: 155-141-2025"); Acknowledgments, p. 32 confirms the sponsor had no role in design, execution, interpretation, or the decision to publish.
Competing interests	26	Declare any competing interests of review authors.	Conflicts of Interest, p. 32: "The authors declare no conflict of interest…"
Availability of data, code and other materials	27	Report which of the following are publicly available and where they can be found: template data collection forms; data extracted from included studies; data used for all analyses; analytic code; any other materials used in the review.	Data Availability Statement, p. 32 (currently: "All data supporting the findings of this review are available within the article and cited references." To be updated per the editor's request to: "No new data were created or analyzed in this study. Data sharing is not applicable.").

Section and Topic	Item #	Checklist item	Location where item is reported
			Underlying source data also provided in Supplementary Tables S3–S5.

29 From: Page MJ, McKenzie JE, Bossuyt PM, Boutron I, Hoffmann TC, Mulrow CD, et al. The PRISMA 2020 statement: an updated guideline for reporting systematic reviews. BMJ 2021;372:n71. doi: 10.1136/bmj.n71. This work is licensed under CC BY 4.0. To view a copy of this license, visit https://creativecommons.org/licenses/by/4.0

Supplementary Table S2. Database-specific search strategies.

Source	Limits / field strategy	Search terms
PubMed/MEDLINE	Title/Abstract search; publication date 1 January 2015 to 31 March 2026; English-language full texts where available.	("chimeric antigen receptor"[Title/Abstract] OR "CAR T"[Title/Abstract] OR "CAR-T"[Title/Abstract] OR "CAR T cell"[Title/Abstract] OR "CAR-T cell"[Title/Abstract] OR "CAR T cells"[Title/Abstract] OR "CAR-T cells"[Title/Abstract] OR "CAR T-cell therapy"[Title/Abstract] OR "CAR-T-cell therapy"[Title/Abstract] OR "CAR Treg"[Title/Abstract] OR "CAR-Treg"[Title/Abstract] OR "regulatory CAR T"[Title/Abstract] OR "engineered T cell"[Title/Abstract]) AND ("autoimmune disease"[Title/Abstract] OR autoimmunity[Title/Abstract] OR "systemic lupus erythematosus"[Title/Abstract] OR SLE[Title/Abstract] OR "lupus nephritis"[Title/Abstract] OR "systemic sclerosis"[Title/Abstract] OR scleroderma[Title/Abstract] OR "rheumatoid arthritis"[Title/Abstract] OR "multiple sclerosis"[Title/Abstract] OR "type 1 diabetes"[Title/Abstract] OR myositis[Title/Abstract] OR "myasthenia gravis"[Title/Abstract] OR infection[Title/Abstract] OR "infectious disease"[Title/Abstract] OR "persistent infection"[Title/Abstract] OR HIV[Title/Abstract] OR AIDS[Title/Abstract] OR "viral reservoir"[Title/Abstract] OR "hepatitis B"[Title/Abstract] OR HBV[Title/Abstract] OR "hepatitis C"[Title/Abstract] OR HCV[Title/Abstract] OR cytomegalovirus[Title/Abstract] OR CMV[Title/Abstract] OR "Epstein-Barr virus"[Title/Abstract] OR EBV[Title/Abstract] OR tuberculosis[Title/Abstract]) NOT (cancer[Title/Abstract] OR tumor[Title/Abstract] OR tumour[Title/Abstract] OR malignancy[Title/Abstract] OR leukemia[Title/Abstract] OR lymphoma[Title/Abstract] OR myeloma[Title/Abstract])
Scopus	TITLE-ABS-KEY search adapted from PubMed terms; publication date 2015 to March 2026; document types screened for original clinical,	TITLE-ABS-KEY(("chimeric antigen receptor" OR "CAR T" OR "CAR-T" OR "CAR T cell" OR "CAR-T cell" OR "CAR Treg" OR "CAR-Treg") AND ("autoimmune disease" OR autoimmunity OR "systemic lupus erythematosus" OR SLE OR "systemic sclerosis" OR "rheumatoid arthritis" OR

Source	Limits / field strategy	Search terms
	preclinical, and translational evidence.	"multiple sclerosis" OR "type 1 diabetes" OR HIV OR HBV OR HCV OR CMV OR EBV OR tuberculosis OR "persistent infection") AND NOT (cancer OR tumor OR tumour OR malignancy OR leukemia OR lymphoma OR myeloma))
Web of Science	Topic search adapted from PubMed terms; publication date 2015 to March 2026.	TS=(("chimeric antigen receptor" OR "CAR T" OR "CAR-T" OR "CAR T cell" OR "CAR-T cell" OR "CAR Treg" OR "CAR-Treg") AND (autoimmune OR "systemic lupus erythematosus" OR "lupus nephritis" OR "systemic sclerosis" OR "rheumatoid arthritis" OR "multiple sclerosis" OR HIV OR HBV OR HCV OR CMV OR EBV OR tuberculosis OR "persistent infection") NOT (cancer OR tumor OR malignancy OR leukemia OR lymphoma OR myeloma))
Cochrane Library	Keyword search for trials and controlled studies.	("CAR T" OR "CAR-T" OR "chimeric antigen receptor") AND (autoimmune OR infection OR HIV OR HBV OR HCV OR CMV OR EBV OR tuberculosis)
ClinicalTrials.gov	Registry search for ongoing or completed trials.	Condition/disease: autoimmune disease OR systemic lupus erythematosus OR rheumatoid arthritis OR multiple sclerosis OR HIV OR hepatitis B OR hepatitis C; Other terms: CAR T OR CAR-T OR chimeric antigen receptor OR CAR-Treg
WHO ICTRP / other trial registries	Registry search adapted from ClinicalTrials.gov.	CAR T OR CAR-T OR chimeric antigen receptor OR CAR-Treg combined with autoimmune disease, HIV, HBV, HCV, CMV, EBV, tuberculosis, or persistent infection terms.
Manual reference screening	Backward and forward citation checking.	Reference lists of included studies and relevant recent reviews were screened manually to identify additional eligible original studies.

Note: Searches were designed for sensitivity because terminology for CAR T-cell and CAR-Treg therapy is not standardized across databases. Database syntax was adapted database syntax to each platform's requirements.

Supplementary Table S3. Aggregate source data summary for Figure 4: disease distribution counts.

Disease domain	Disease indication	Studies, *n*	Included in Figure 2	Interpretation
Autoimmune disease	Systemic lupus erythematosus (SLE)	14	Yes	Largest autoimmune evidence group; strongest early clinical signal for B-cell-directed CAR T-cell therapy.
Autoimmune disease	Rheumatoid arthritis (RA)	8	Yes	Smaller and more heterogeneous evidence base than SLE.
Autoimmune disease	Multiple sclerosis (MS)	6	Yes	Early and mixed evidence; neurological endpoints require longer follow-up.
Autoimmune disease	Systemic sclerosis (SSc)	5	Yes	Promising organ-specific response signal, but evidence remains preliminary.

Disease domain	Disease indication	Studies, *n*	Included in Figure 2	Interpretation
Autoimmune disease	Type 1 diabetes (T1D)	3	Yes	Mainly early regulatory/tolerance-focused evidence.
Autoimmune disease	Inflammatory myopathies	2	Yes	Very small evidence base; interpreted descriptively.
Infectious disease	HIV/AIDS	12	Yes	Largest infectious disease group; evidence focused on antiviral activity, reservoir targeting, and CAR persistence.
Infectious disease	Hepatitis B virus (HBV)	6	Yes	Evidence focused on HBV antigen targeting and functional-cure concepts.
Infectious disease	Hepatitis C virus (HCV)	5	Yes	Limited and confounded evidence where antiviral retreatment is used.
Infectious disease	Cytomegalovirus (CMV) infection	3	Yes	Small evidence base in immunocompromised settings.
Infectious disease	Epstein-Barr virus (EBV)-associated disease	2	Yes	Early evidence; small number of studies.
Infectious disease	Tuberculosis (TB)	1	Yes	Feasibility-level evidence only.
Qualitative/mechanistic/platform evidence	Cross-disciplinary or contextual evidence	11	No	Contributed mechanistic, platform-related, or contextual evidence but was not included in disease-count distribution.
Total	Structured descriptive analysis	67	Yes	38 autoimmune disease studies and 29 infectious disease studies.
Total	Final qualitative synthesis	78	Partly	67 studies contributed to structured descriptive analysis; 11 contributed qualitative/mechanistic/contextual evidence.

Note: Values are descriptive study counts and are not pooled effect estimates.

Supplementary Table S4A. Aggregate source data summary for Figure 4: CAR T-cell construct generation distribution.

CAR T-cell construct generation	Autoimmune disease studies, n (%)	Infectious disease studies, *n* (%)	Total studies, *n* (%)	Interpretation
First-generation CAR, CD3ζ only	2 (5.3%)	3 (10.3%)	5 (7.5%)	Early proof-of-concept constructs; limited persistence.
Second-generation CAR, CD28 co-stimulation	10 (26.3%)	8 (27.6%)	18 (26.9%)	Used where rapid effector function or established second-generation design was prioritized.
Second-generation CAR, 4-1BB co-stimulation	14 (36.8%)	7 (24.1%)	21 (31.3%)	Most frequent construct group overall; interpreted as favoring persistence and sustained activity.
Third-generation CAR, dual co-stimulation	5 (13.2%)	4 (13.8%)	9 (13.4%)	Designed to enhance activation through dual costimulatory signaling.

CAR T-cell construct generation	Autoimmune disease studies, n (%)	Infectious disease studies, *n* (%)	Total studies, *n* (%)	Interpretation
Fourth-generation armored/TRUCK or cytokine-armed CAR	5 (13.2%)	4 (13.8%)	9 (13.4%)	Designed to enhance immune modulation, trafficking, persistence, or resistance to inhibitory environments.
Universal/allogeneic CAR T platform	2 (5.3%)	3 (10.3%)	5 (7.5%)	Early off-the-shelf approaches; still under investigation.
Total	38 (100%)	29 (100%)	67 (100%)	Descriptive construct-distribution denominator for Figure 3.

Note: Studies were classified according to the predominant CAR T-cell construct generation or platform described. Values are descriptive counts and percentages, not inferential comparisons.

Supplementary Table S4B. Representative construct designs and disease-specific rationale in non-oncological CAR T-cell therapy.

Disease/application area	Target antigen or target system	Representative CAR design	Binding-domain logic	Costimulatory/platform logic	Why this design is suited to the disease context	Main limitation
SLE / lupus nephritis	CD19	CD19-directed CAR T cell	Targets broad B-cell developmental compartments	Usually second-generation CD28 or 4-1BB CAR	Enables deep B-cell depletion, reduction of autoreactive memory B cells, and possible immune reset	Small cohorts; relapse, long-term hypogammaglobulinemia, and infection risk remain uncertain
SLE / lupus nephritis	BCMA	BCMA-directed CAR T cell	Targets plasmablasts and plasma cells	Persistent cytotoxic CAR platform	May deplete autoantibody-producing compartments not fully removed by CD20-directed therapies	Risk of prolonged plasma-cell depletion, hypogammaglobulinemia, and infection
Systemic sclerosis / inflammatory myopathies	CD19	CD19-directed CAR T cell	Targets B cells involved in autoantibody production, antigen presentation, and cytokine amplification	Second-generation CAR platforms	May interrupt immune-fibrotic or immune-myopathic inflammatory networks	Very small cohorts; organ-specific endpoints not standardized
Rheumatoid arthritis /	CD19 or CD20-oriented B-cell targeting	B-cell-directed CAR T cell	Targets pathogenic B-cell contribution	Second-generation or	May reduce autoreactive B-cell activity and	Evidence less mature than SLE;

Disease/application area	Target antigen or target system	Representative CAR design	Binding-domain logic	Costimulatory/platform logic	Why this design is suited to the disease context	Main limitation
multiple sclerosis			to autoimmunity	experimental constructs	inflammatory antigen presentation	neurological and joint endpoints require longer follow-up
Type 1 diabetes / IBD / tolerance applications	Disease-relevant tissue antigen	CAR-Treg	Antigen-specific localization of regulatory T cells	Regulatory-cell platform prioritizing FOXP3 stability and suppressive function	Supports localized immune tolerance rather than systemic immunosuppression	Lineage instability, inflammatory conversion, and manufacturing complexity
HIV infection	HIV Env / gp120	CD4-derived or envelope-targeted CAR T cell	Recognizes HIV envelope-expressing infected cells	Early first- or second-generation CAR designs	Provides virus-specific recognition and proof-of-concept antiviral activity	Viral escape, low antigen expression, reservoir accessibility, and possible infection of the CAR T product
HIV infection	Conserved Env epitopes	bNAb-derived or dual-targeting CAR T cell	Uses broadly neutralizing antibody-derived recognition or multispecific targeting	Dual-targeting/protected platforms; possible CCR5 disruption	May broaden recognition, reduce escape, and protect engineered cells	Durable reservoir eradication not proven
HBV infection	HBsAg / HBV envelope antigens	HBV antigen-directed CAR T cell	Recognizes HBV antigen-expressing infected cells	Preferably transient, safety-controlled, or dose-controllable design	May support infected-cell targeting or functional-cure strategies	On-target liver injury; infected hepatocytes are non-expendable
HCV infection	HCV E2	Anti-E2 CAR T cell	Targets viral envelope antigen	Experimental antiviral CAR platform	Provides proof-of-concept pathogen targeting	Confounded by effective direct-acting antivirals; limited clinical need
CMV infection	pp65 or CMV-associated antigen system	CMV-directed CAR T cell	Targets CMV-associated viral antigen	Pathogen-directed cellular therapy platform	May support antiviral control in immunocompromised patients	Small evidence base; antigen accessibility and immune status vary

Disease/application area	Target antigen or target system	Representative CAR design	Binding-domain logic	Costimulatory/platform logic	Why this design is suited to the disease context	Main limitation
EBV-associated disease	LMP1 or EBV-associated antigen	EBV-directed CAR T cell	Targets EBV-associated infected or transformed cells	Pathogen-directed cytotoxic CAR platform	May support control of EBV-associated disease in selected settings	Small cohorts; overlap with lymphoproliferative disease biology
Transient immune modulation	Disease-specific antigen	mRNA CAR T cell / short-lived CAR expression / safety-switch CAR	Temporary antigen-directed recognition	Reversible or controllable CAR expression	Useful where prolonged depletion or tissue injury is undesirable	May require repeat dosing; durability may be limited

Note: This table supports biological interpretation and does not imply that all constructs have equivalent clinical maturity or efficacy.

Supplementary Table S5A. Descriptive source-data summary for Figure 5: response endpoints by disease indication and CAR T-cell target.

Disease indication	Disease domain	CAR target/platform	Contributing studies, *n*	Evaluable sample size, *n*	Response endpoint used in descriptive synthesis	Descriptive response estimate shown in Figure 4
SLE	Autoimmune	CD19 CAR T cells	14	342	Complete remission or clinical remission, serological improvement, complement recovery, anti-dsDNA reduction, and lupus nephritis response where applicable	92 [85-97]
SLE/lupus nephritis subset	Autoimmune	BCMA CAR T cells	3	47	Response in refractory lupus nephritis or plasma-cell-directed response	87 [78-94]
RA	Autoimmune	CD19 CAR T cells	8	186	Overall response, ACR response, or DAS28-CRP improvement as defined by original studies	78 [65-88]
MS	Autoimmune	CD19/CD20 CAR T cells	6	94	Annualized relapse-rate reduction, NEDA-3, or MRI lesion reduction	72 [60-82]
SSc	Autoimmune	CD19 CAR T cells	5	98	Modified Rodnan Skin Score improvement or organ-specific response	68 [54-80]
T1D	Autoimmune	CAR-Treg/islet-antigen-directed platform	3	45	C-peptide preservation or reduced insulin requirement	55 [38-70]
Inflammatory myopathies	Autoimmune	CD19 CAR T cells	2	28	Creatine kinase normalization and functional improvement	Not plotted separately

Disease indication	Disease domain	CAR target/platform	Contributing studies, *n*	Evaluable sample size, *n*	Response endpoint used in descriptive synthesis	Descriptive response estimate shown in Figure 4
HIV/AIDS	Infectious	Anti-gp120/CD4-derived CAR T cells	6	142	HIV DNA reduction, viral load response, or antiviral activity as defined by original studies	64 [51-75]
HIV/AIDS	Infectious	bNAb-derived or dual-targeting CAR T cells	6	142	Antiviral activity, reservoir response, delayed rebound, or ART-free suppression were reported	71 [58-82]
HBV	Infectious	Anti-HBsAg/HBV envelope-directed CAR T cells	6	156	HBsAg loss, HBV DNA suppression, or functional-cure-related endpoint	58 [42-72]
HCV	Infectious	Anti-E2 CAR T cells	5	108	Sustained virologic response or viral clearance	52 [36-66]
CMV infection	Infectious	Anti-pp65 CAR T cells	3	62	CMV clearance in immunocompromised recipients	74 [59-86]
EBV-associated disease	Infectious	Anti-LMP1 CAR T cells	2	38	Objective response or disease response in EBV-associated disease	62 [45-77]
TB	Infectious	Anti-Ag85B CAR T cells	1	8	Immunological enhancement or microbiological response	Not plotted separately

Note: Response endpoints were extracted according to definitions reported by the original studies and were not harmonized into a single universal remission endpoint. Bracketed values represent descriptive reported intervals or ranges used for visualization, not formal pooled confidence intervals. Direct comparison between diseases or CAR targets is not appropriate.

Supplementary Table S5B. Descriptive source data summary for Figure 6: safety reporting frequencies by adverse event category and disease domain.

Adverse-event category	Autoimmune studies reporting, n/N (%)	Infectious disease studies reporting, n/N (%)	Patient-level denominator availability	Clinical/preclinical interpretation
CRS, any grade	26/38 (68%)	13/29 (45%)	Patient-level denominators not consistently extractable across all studies	Clinical safety signal; most commonly reported acute toxicity.
CRS, grade >=3	3/38 (8%)	3/29 (12%)	Patient-level denominators not consistently extractable across all studies	Clinical safety signal; severe CRS was uncommon.
ICANS/neurotoxicity	2/38 (5%)	2/29 (8%)	Patient-level denominators not consistently extractable across all studies	Clinical safety signal; neurotoxicity was uncommon where reported.

Adverse-event category	Autoimmune studies reporting, n/N (%)	Infectious disease studies reporting, n/N (%)	Patient-level denominator availability	Clinical/preclinical interpretation
Cytopenias	16/38 (42%)	11/29 (38%)	Patient-level denominators not consistently extractable across all studies	Clinical safety signal; often associated with lymphodepleting conditioning.
Infectious complications	11/38 (28%)	10/29 (35%)	Patient-level denominators not consistently extractable across all studies	Clinical safety signal; includes respiratory infections and viral reactivation where reported.
Hypogammaglo bulinemia	20/38 (52%)	9/29 (30%)	Patient-level denominators not consistently extractable across all studies	Clinical safety signal; more frequent in B-lineage-targeted autoimmune studies.
B-cell aplasia	25/38 (65%)	8/29 (28%)	Patient-level denominators not consistently extractable across all studies	Expected on-target effect of CD19-directed CAR T-cell therapy.
Hepatic flares / liver enzyme elevation	Not consistently reported	Reported mainly in HBV-directed studies	Patient-level denominators not consistently extractable across all studies	Disease-specific safety concern in HBV-directed CAR T-cell therapy because the liver is a non-expendable target organ.
Treatment-related mortality	0/38 (0%)	0/29 (0%)	No treatment-related deaths reported in the descriptive synthesis	Clinical safety signal; no grade 5 treatment-related events were reported.

Note: Values represent study-level reporting frequencies within each disease domain and should not be interpreted as pooled patient-level adverse-event incidence rates. Clinical studies were prioritized for patient-safety interpretation. Preclinical, in vitro, translational, and platform-development studies were used only to support mechanistic safety considerations and were not used to infer clinical toxicity rates. CRS, cytokine release syndrome; HBV, hepatitis B virus; ICANS, immune effector cell-associated neurotoxicity syndrome.
